# Betaine-driven Keap1 methylation promotes p62-mediated autophagy and Nrf2 stabilization to alleviate glutathione-dependent redox dyshomeostasis in liver injury

**DOI:** 10.1186/s43556-026-00579-1

**Published:** 2026-09-16

**Authors:** Moon Han Chang, Chae Hwan Lee, Yeongrae Cho, Ryeo Gang Son, Seoyoon Lee, Shinwoo Park, Jaeryeong Kim, Young Ho Koh, Art E. Cho, Seung Pil Pack, Minseok Seo, Jin Hyup Lee, Hanvit Cha

**Affiliations:** 1 Division of Brain Disease Research, Department of Chronic Disease Convergence Research, Korea National Institute of Health, Cheongju, Republic of Korea; 2 Department of Food and Biotechnology, Korea University, Sejong, Republic of Korea; 3 BK21 FOUR Research Group for Omics-Based Bio-Health in Food Industry, Korea University, Sejong, Republic of Korea; 4 Biological Clock-based Anti-aging Convergence RLRC, Korea University, Sejong, Republic of Korea; 5 Department of Biotechnology and Bioinformatics, Korea University, Sejong, Republic of Korea; 6 Advanced Drug Quality Division Department, National Institute of Food and Drug Safety Evaluation, Ministry of Food and Drug Safety, Cheongju, Republic of Korea; 7 Department of Computer and Information Science, Korea University, Sejong, Republic of Korea; 8 Institutes of Natural Sciences, Korea University, Sejong, Republic of Korea

**Keywords:** Betaine, Keap1 methylation, p62, Nrf2, Liver injury

## Abstract

Nuclear factor erythroid 2–related factor 2 (Nrf2) coordinates detoxification and antioxidant programs that protect the liver from chemical and metabolic stress. Nrf2 is restrained by Kelch-like ECH-associated protein 1 (Keap1), which can be removed through p62-mediated selective autophagy; however, nutritional cues that engage this pathway remain incompletely understood. This study provides evidence that betaine (trimethylglycine; BET) can regulate the Keap1-Nrf2 axis through modulation of Keap1. In biochemical and cellular assays, betaine was associated with methylation of Keap1 at Cys513 and Cys518 within the Kelch domain, which was linked to enhanced p62-dependent autophagic degradation of Keap1 and Nrf2 stabilization. In AML12 hepatocytes and in mice, betaine enhanced Nrf2 activity, induced canonical Nrf2 target genes, and increased cellular glutathione (GSH), consistent with improved GSH-dependent redox capacity. In an acetaminophen (AAP) overdose model, betaine attenuated hepatic injury, consistent with improved redox capacity under acute toxic stress. In an MCD diet-induced steatotic liver disease model, betaine was associated with reduced steatohepatitis-like histopathology, inflammatory signaling, and lipogenic markers. Finally, RNA-seq analysis of a large human metabolic dysfunction-associated steatotic liver disease (MASLD) cohort revealed Nrf2-related transcriptomic signatures associated with disease severity, providing correlative support for the disease relevance of this pathway. Collectively, these findings suggest that Keap1 cysteine methylation may represent a regulatory node linking betaine to p62-mediated autophagy, Nrf2 stabilization, and GSH homeostasis, while further studies are required to establish the causal and translational relevance of this mechanism in human liver disease.

**Supplementary Information:**

The online version contains supplementary material available at https://doi.org/10.1186/s43556-026-00579-1.

## Introduction

Cells and organisms are continuously challenged by endogenous and exogenous chemical stressors, including toxicants and reactive oxygen species (ROS), and therefore rely on coordinated detoxifying and antioxidant defense systems to maintain redox homeostasis [1, 2]. Nrf2 is a transcription factor that counteracts redox dyshomeostasis, defined here as a disruption of redox homeostasis in which oxidant burden (e.g., ROS) exceeds antioxidant capacity (e.g., GSH), leading to oxidative stress. Although Nrf2 is ubiquitously expressed, Keap1 normally sequesters it in the cytosol and targets it for degradation under basal conditions. During redox dyshomeostasis, Keap1-dependent repression is relieved, allowing Nrf2 to accumulate and translocate to the nucleus, where it induces cytoprotective genes, including antioxidant and phase 2 detoxification enzymes [2, 3].

Various post-translational modifications (PTMs) of Keap1 have been implicated in the regulation of the Keap1-Nrf2 signaling pathway. Specifically, chemical modifications of Keap1 cysteine residues, such as S-nitrosylation and oxidation, can contribute to Nrf2 pathway activation by altering Keap1-mediated repression [4]. The highly reactive cysteine residues in Keap1 sense electrophiles, including toxicants and ROS, thereby activating the Nrf2 signaling pathway. The thiol groups of Keap1 cysteine residues enable electrophile sensing and may influence Keap1 regulation, including p62-mediated autophagic degradation under specific cellular contexts [3, 5, 6]. Numerous electrophilic drugs that modulate the Keap1-Nrf2 signaling pathway have been developed as therapeutic targets for diseases associated with redox dyshomeostasis [7]. Consequently, there is a growing interest in developing novel therapeutic drugs targeting Keap1 for the treatment of various diseases linked to disruption in redox homeostasis [8].

Trimethylglycine, also known as betaine (BET), is found in microorganisms, plants, and animals, and is an abundant nutrient in foods such as spinach, wheat, and sugar beets. In humans, betaine is primarily found in the kidneys and liver, where it participates in methyl metabolism as a methyl donor due to its electrophilic methyl groups [9]. One-carbon metabolism plays a significant role in GSH synthesis by generating homocysteine and serine, which serve as precursors of glycine and cysteine, essential building blocks of GSH [10]. In vitro studies suggest that the antioxidant properties of betaine are primarily associated with GSH synthesis rather than direct ROS scavenging [11]. Human studies have reported associations among choline, dimethylglycine (DMG), betaine, and glycine-related metabolism, suggesting a potential link to GSH synthesis [12]. Beyond its metabolic role, betaine has also shown potential for epigenetic regulation through histone H3 methylation [13]. Recently, betaine administration has been intriguingly reported to upregulate genes associated with GSH synthesis [14]. However, the underlying mechanism regulating the transcription of these cytoprotective genes remains unclear.

In this study, we hypothesized that betaine promotes p62-mediated autophagic degradation of Keap1 by methylating specific cysteine residues in its Kelch domain (Cys513 and Cys518), thereby stabilizing Nrf2 and enhancing GSH-dependent redox homeostasis. To test this, we performed mechanistic analyses in vitro to define how betaine modifies Keap1 and activates Nrf2 signaling. We identified the relevant cysteine residues using liquid chromatography–mass spectrometry (LC–MS), site-directed mutagenesis, and molecular docking. The proposed mechanism was then evaluated in vivo, supporting the association of betaine treatment with enhanced Keap1 turnover and induction of Nrf2 target genes linked to GSH homeostasis. Finally, disease relevance was assessed in murine models of AAP-induced acute liver injury and MCD diet–induced steatotic liver disease, and clinical consistency was examined using publicly available RNA-seq data from a large human MASLD cohort deposited in the Gene Expression Omnibus (GEO) database under accession number GSE192959. Together, these findings support Keap1 cysteine methylation as a possible mechanistic link between betaine and Nrf2 activation and suggest the need for further evaluation of betaine-related Keap1–Nrf2 regulation in liver diseases driven by GSH-dependent redox dyshomeostasis.

## Results

### Betaine methylates the Cys513 and Cys518 residues of Keap1, promoting its autophagic degradation and subsequent activation of Nrf2 in hepatocytes

**Fig. 1:**
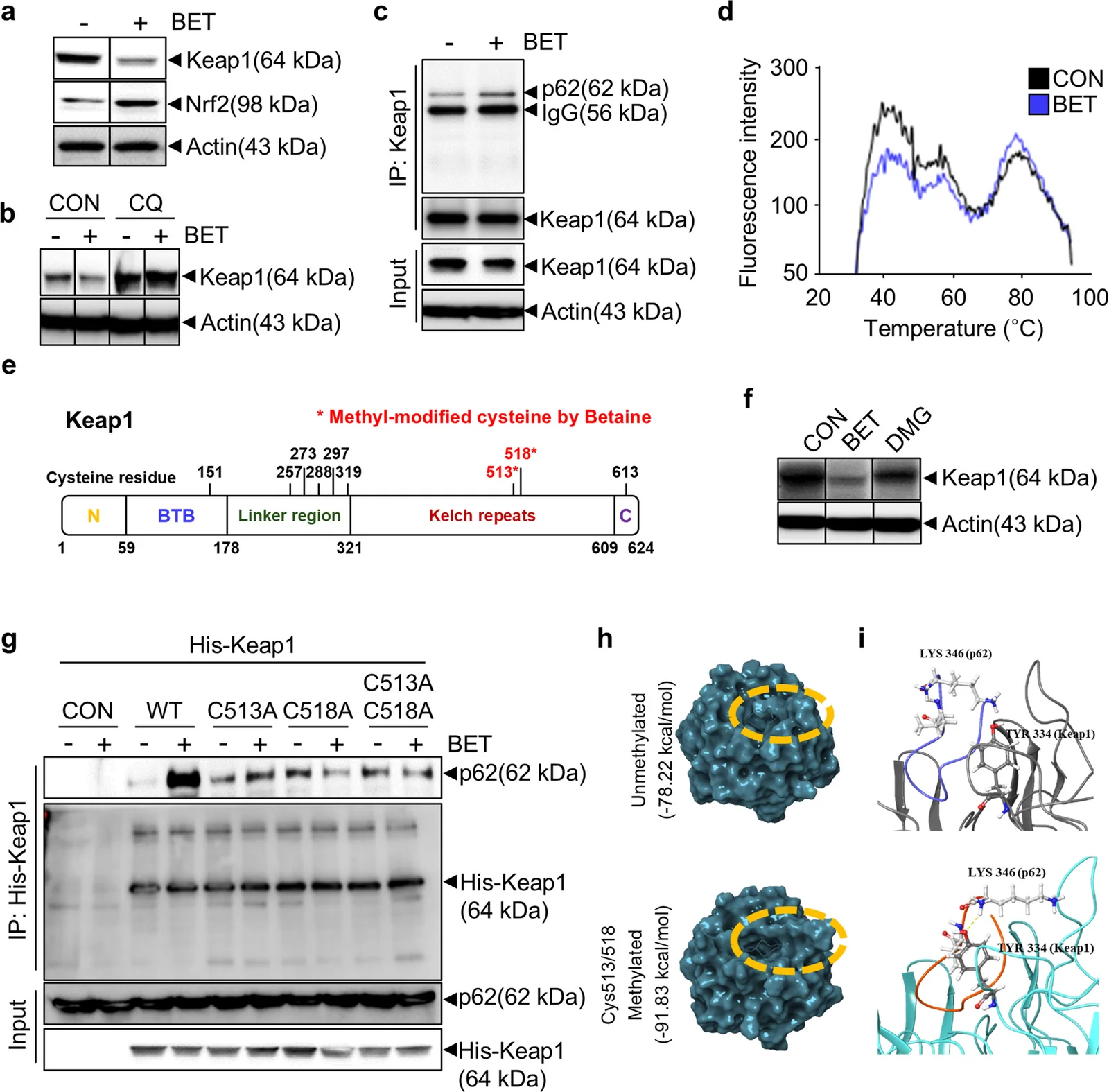
Betaine methylates Keap1 at Cys513 and Cys518 to promote p62-mediated autophagic degradation and Nrf2 stabilization in hepatocytes. AML12 cells were treated with betaine (100 μM) for 3 h unless otherwise indicated. **a** Immunoblot analysis of Keap1 and Nrf2 in AML12 cells. **b** Immunoblot analysis of Keap1 in AML12 cells pretreated with CQ for 24 h prior to betaine treatment. **c** co-IP of Keap1 followed by immunoblotting for p62 and Keap1; the ~50 kDa band corresponds to the IgG heavy chain detected by the secondary antibody. **d** Thermal shift assay showing increased Keap1 thermal stability in the presence of betaine (blue) compared with control (black). **e** Schematic model of Keap1 methylation inferred from LC–MS, indicating methyl transfer to Cys513 and Cys518 within the Kelch domain. **f** Immunoblot analysis of Keap1 in AML12 cells treated with betaine or DMG (100 μM) for 3 h. **g** Immunoblot analysis of p62 and His–Keap1 in AML12 cells transfected with His-tagged Keap1 constructs (WT, C513A, C518A, or C513A/C518A) and treated with betaine. **h** Representative surface structures of wild-type and methylated Keap1 bound to p62. Yellow dashed ellipses indicate the predicted interaction interface between Keap1 and p62. The calculated binding energies for the wild-type and methylated complexes are shown for each structure. **i** Structural comparison indicating the differential interaction between Keap1 Tyr334 and p62 Lys346; corresponding quantification is shown on the right. Quantitative graphs are presented with individual values, mean ± SD, and group labels matching the corresponding panel colors

To determine whether betaine regulates the Keap1–Nrf2 axis through Keap1 degradation and to define the responsible modification, we treated AML12 hepatocytes with betaine with/without chloroquine (CQ) and performed immunoblotting, qPCR, immunofluorescence, co-immunoprecipitation (co-IP), thermal shift assays using purified Keap1, LC–MS, Keap1 cysteine mutagenesis, and molecular dynamics (MD) simulations. Betaine decreased Keap1 protein levels and increased cytosolic and nuclear Nrf2 within 3 h (Fig. 1a; Fig. S1), accompanied by induction of Nrf2 target genes, including Glutathione synthetase (*Gss*), Glutamate-cysteine ligase (*Gclm*), Catalase (*Cat*), Superoxide dismutase 2 (*Sod2*), and Heme oxygenase (*Hmox1*), at both protein and mRNA levels, while Keap1 mRNA remained unchanged (Fig. S2a-c). CQ restored Keap1 levels reduced by betaine (Fig. 1b) and abolished betaine-induced GSS mRNA upregulation (Fig. S3), indicating an autophagy-dependent mechanism. Consistently, co-IP showed increased p62 association with Keap1 upon betaine treatment (Fig. 1c). A thermal shift assay supported direct interaction between betaine and Keap1 (Fig. 1d), and LC–MS identified methylation at Keap1 Cys513 and Cys518 rather than covalent attachment of intact betaine (Fig. 1e; Fig. S4). DMG, which contains one fewer methyl group than betaine, failed to reduce Keap1 levels (Fig. 1f), supporting a requirement for methyl-donor capacity. Betaine enhanced p62 binding to His-tagged wild-type Keap1 but not to C513A, C518A, or C513A/C518A mutants (Fig. 1g), supporting the involvement of Cys513/Cys518 in betaine-associated regulation of the Keap1-p62 interaction. MD simulations of the Keap1 Kelch domain (PDB 3WDZ) further supported strengthened p62 interaction with methylated Keap1, showing more favorable binding energy and a distinct stabilizing contact involving p62 Lys346 and Keap1 Tyr334 (Fig. 1h, i; Fig. S5a–g). Together, these data support a model in which betaine-associated methylation of Keap1 at Cys513/518 may facilitate p62-mediated autophagic Keap1 degradation, thereby contributing to Nrf2 stabilization and activation of antioxidant and GSH-related gene programs.

### Betaine administration enhances Nrf2-mediated GSH redox homeostasis by modulating the autophagic degradation of Keap1 in the liver of mice

**Fig. 2:**
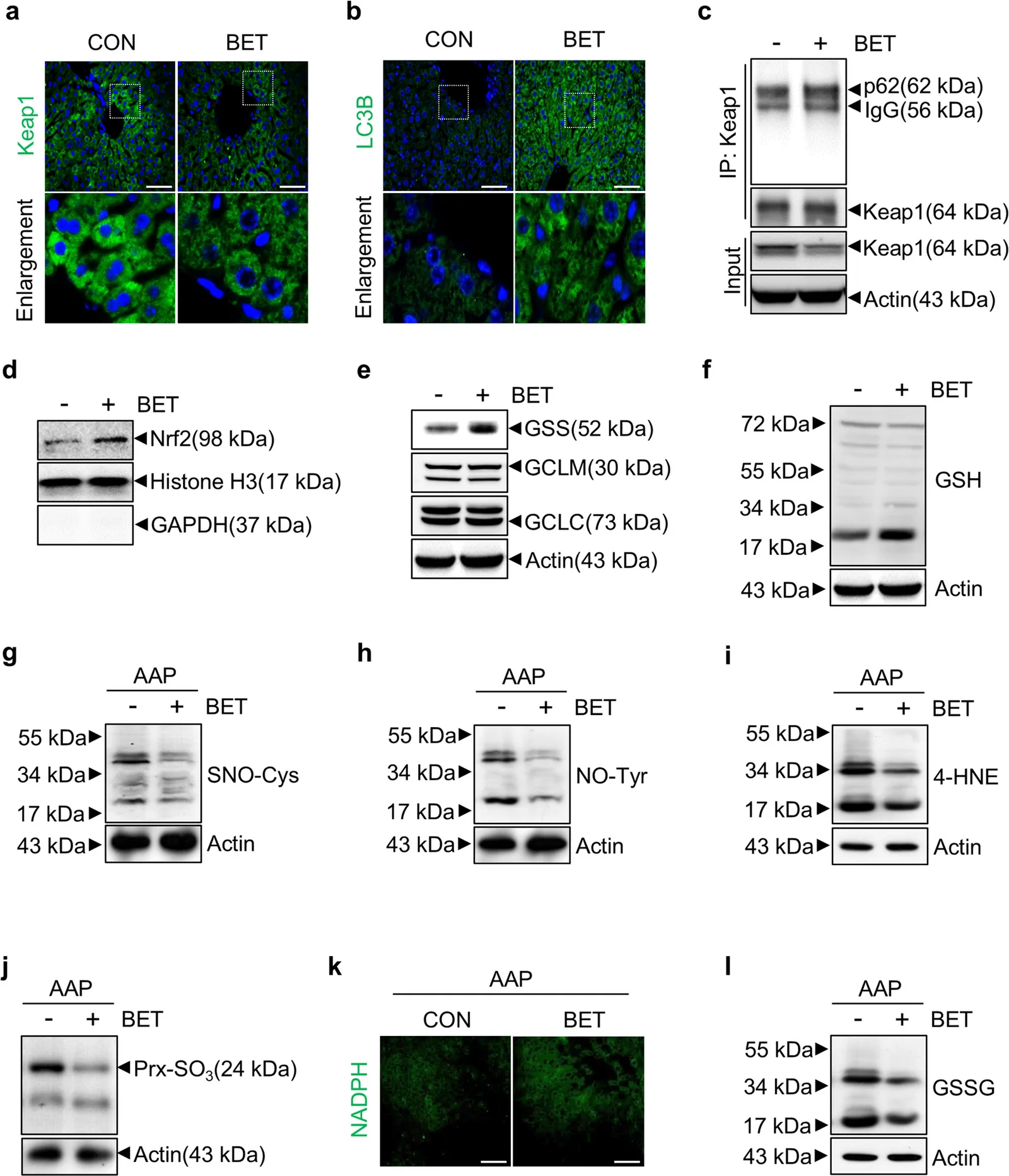
Betaine enhances hepatic Nrf2 signaling and GSH redox homeostasis via autophagy-associated Keap1 degradation in vivo. Mice were orally administered betaine (BET) for 7 days. For betaine-only analyses, panels are presented as CON and BET where applicable. For the AAP-challenged model, mice were challenged with AAP 2 h after the final betaine dose; livers were collected 12 h after AAP. AAP model panels are presented as AAP and AAP + BET where applicable. **a** Representative immunofluorescence images of Keap1 (green) and nuclei counterstained with DAPI (blue) in liver sections (original magnification, 400 ×). Lower panels show magnified views of the boxed regions in the corresponding upper panels. Scale bars, 50 μm (upper panels). **b** Representative immunofluorescence images of LC3B (green) and nuclei counterstained with DAPI (blue) in liver sections (original magnification, 400 ×). Lower panels show magnified views of the boxed regions in the corresponding upper panels. Scale bars, 50 μm (upper panels). **c** Co-IP of Keap1 from liver lysates followed by immunoblotting for p62. **d** Immunoblot analysis of nuclear Nrf2 in liver nuclear fractions; GAPDH was used as a negative control for nuclear extraction. **e** Immunoblot analysis of GSS, GCLM, and GCLC in liver lysates. **f** Hepatic GSH levels. For the AAP model, mice were orally administered betaine for 7 days and challenged with AAP 2 h after the final betaine dose; livers were collected 12 h after AAP. Immunoblot analysis of **g** SNO-Cys, **h** NO-Tyr, **i** 4-HNE, and **j** Prx-SO3 in liver lysates. **k** Representative immunofluorescence images of NADPH (green) in liver Sections. (200 ×; scale bar, 100 μm). The single-channel images are shown using the original acquisition colors, with channel identities indicated in each panel. **l** Immunoblot analysis of GSSG in liver lysates. Data are presented as mean ± SD with individual values shown and were analyzed by Student’s t-test; for cell-based experiments, *n* = 3 independent experiments, and for animal studies, *n* = 5 mice per group; *P* < 0.05

To assess organ-level regulation, mice received oral betaine for 7 days and liver tissues were compared with untreated controls. Immunofluorescence staining showed reduced Keap1 signal in hepatocytes in the betaine group (Fig. 2a). Consistent with autophagy-dependent Keap1 turnover, LC3B signal was increased in hepatocytes (Fig. 2b), and co-IP of liver extracts revealed increased p62 association with Keap1 (Fig. 2c). Nrf2 activation was further supported by elevated nuclear Nrf2 levels in betaine-treated livers (Fig. 2d). Among Nrf2-responsive GSH-related enzymes, GSS protein was increased, whereas GCLM and GCLC were unchanged (Fig. 2e), and hepatic GSH content was significantly higher in the betaine group (Fig. 2f). To test whether this reinforced redox capacity protects against GSH-depletion–driven oxidative injury, we employed an acetaminophen (AAP; 500 mg/kg) overdose model under the betaine treatment regimen and compared AAP-only versus AAP + betaine groups. Betaine significantly reduced oxidative and nitrosative stress markers, including S-nitroso-cysteine (SNO-Cys), Nitrotyrosine (NO-Tyr), and 4-hydroxynonenal (4-HNE) adducts (Fig. 2g–i), and decreased Hyperoxidized Peroxiredoxin (Prx-SO3), a marker of severe oxidative stress (Fig. 2j). In parallel, betaine increased hepatic Nicotinamide adenine dinucleotide phosphate (NADPH) levels, as shown by immunofluorescence imaging and quantification (Fig. 2k; Fig. S6a), reduced hepatic Glutathione disulfide (GSSG) levels relative to AAP alone (Fig. 2l), and restored depleted hepatic GSH levels (Fig. S6b). Together, these results indicate that betaine promotes autophagy-associated Keap1 degradation and Nrf2 activation in mouse liver, thereby improving GSH redox homeostasis and mitigating AAP-induced oxidative stress.

### Betaine attenuates AAP-induced acute liver injury in association with improved GSH-dependent redox homeostasis and Keap1-Nrf2 pathway activation

**Fig. 3:**
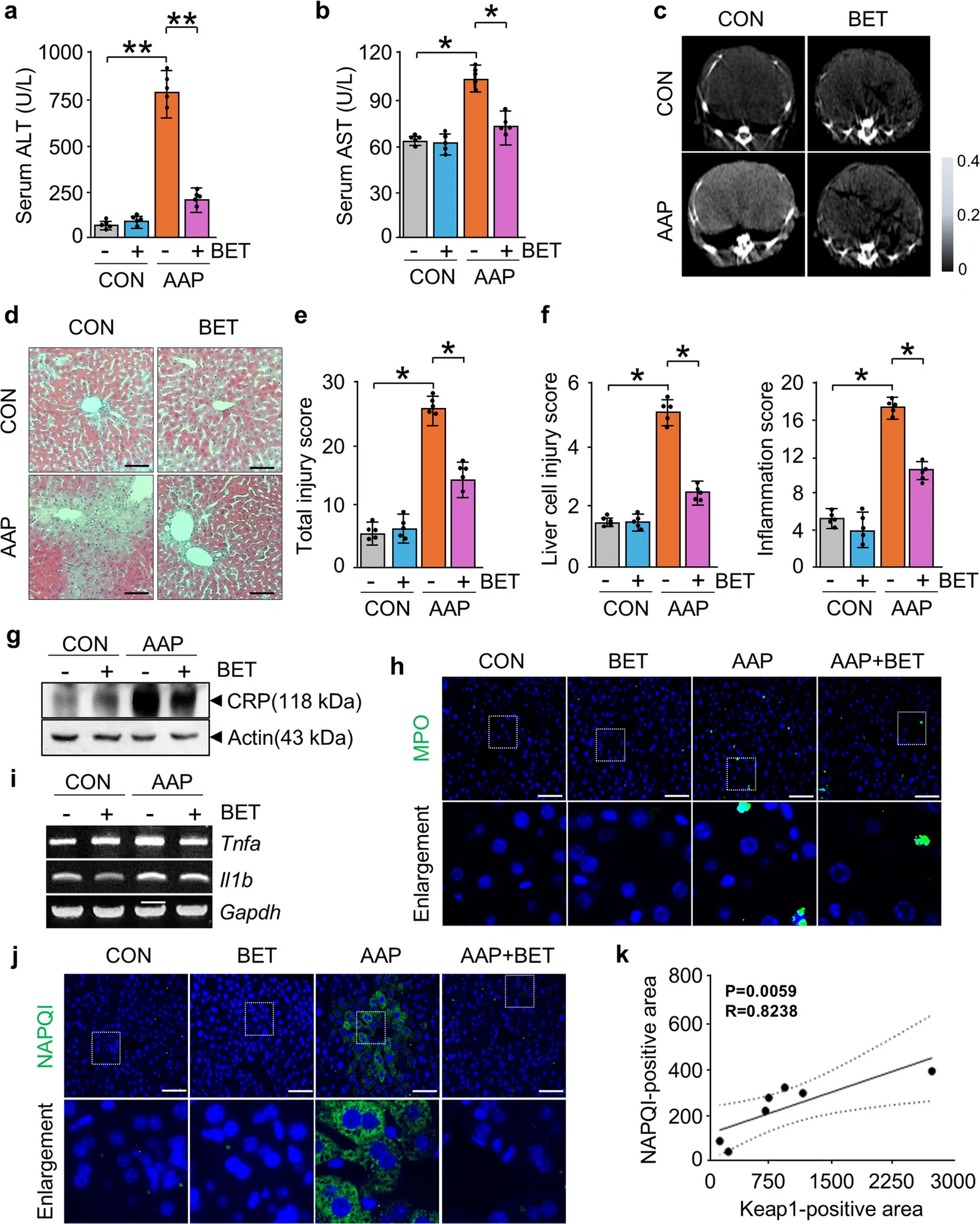
Betaine attenuates AAP-induced acute liver injury and inflammation and is associated with activation of the Keap1–Nrf2 axis. Mice were assigned to four groups: CON, BET, AAP, and AAP + BET. Betaine was orally administered for 7 days, and AAP was administered 2 h after the final betaine dose; samples were collected 12 h after AAP challenge. Unless otherwise indicated, figure panels are presented in the following group order: CON, BET, AAP, and AAP + BET. For panels labeled with −/+, the symbols denote the absence (−) or presence (+) of the indicated treatment under the corresponding experimental condition. **a**, **b** Serum ALT and AST levels. **c** Representative grayscale micro–CT images of liver regions showing altered tissue density. The grayscale intensity scale indicates relative tissue density/intensity. **d** Representative H&E-stained liver sections from each experimental group (200 ×; scale bar, 100 μm). **e**, **f** Histological scoring of total injury, hepatocellular injury, and inflammation based on H&E sections. **g** Immunoblot analysis of CRP in liver lysates. **h** Representative immunofluorescence images of MPO (green) and nuclei counterstained with DAPI (blue) in liver sections (original magnification, 400 ×). Lower panels show magnified views of the boxed regions in the corresponding upper panels. Scale bars, 50 μm (upper panels). **i** Conventional PCR analysis of hepatic TNF-α and IL-1β, with GAPDH as the reference gene. **j** Representative immunofluorescence images of NAPQI (green) and nuclei counterstained with DAPI (blue) in liver sections (original magnification, 400 ×). Lower panels show magnified views of the boxed regions in the corresponding upper panels. Scale bars, 50 μm (upper panels). **k** Pearson correlation between Keap1 signal and hepatic NAPQI levels in AAP-treated groups (*n* = 7). Quantification is shown to the right where indicated. Data are presented as mean ± SD with individual values shown and were analyzed by Student’s t-test; *n* = 5 mice per group unless otherwise stated; *P* < 0.05

After establishing that betaine enhanced hepatic Nrf2 signaling and GSH-dependent redox homeostasis under an AAP-induced acute redox-stress challenge, we next assessed whether these molecular changes were associated with improved pathological outcomes in vivo. To determine whether the redox-protective effects observed in the AAP model translated into improved liver injury outcomes, we compared control (CON), betaine-only (BET), AAP, and AAP + betaine (AAP + BET) groups using biochemical, imaging, histological, and inflammatory endpoints. Serum alanine aminotransferase (ALT) and aspartate aminotransferase (AST) were markedly increased after AAP challenge and were significantly reduced by betaine co-treatment. The BET group showed no evidence of liver injury relative to control, supporting tolerability under the dosing regimen (Fig. 3a, b). In contrast, DMG co-administration did not reduce serum ALT or AST in the AAP model (Fig. S7a, b), supporting a betaine-specific protective effect. Micro-CT imaging revealed increased liver tissue density in AAP-treated mice, which was attenuated in the AAP + BET group (Fig. 3c; Fig. S8a). Consistently, H&E staining showed higher total injury, hepatocellular injury, and inflammation scores in the AAP group relative to Control, whereas these histological scores were significantly improved by betaine (Fig. 3d–f). Inflammatory readouts mirrored these changes: C-reactive protein (CRP) was elevated by AAP and decreased with betaine (Fig. 3g), MPO-positive infiltrating cells were increased by AAP and reduced in AAP + BET livers (Fig. 3h), and pro-inflammatory mediators were attenuated by betaine, including reduced hepatic *Tnf* and *Il1b* mRNA, encoding TNF-α and IL-1β, respectively (Fig. 3i) and decreased IL-6 protein (Fig. S8b). BET group also lowered hepatic NAPQI levels compared with AAP alone (Fig. 3j), without altering CYP2E1 expression (Fig. S8c). To further support the mechanistic interpretation of the main injury phenotypes, additional pathway analyses were performed in liver tissues. These supplementary data showed that Keap1 signal in hepatocytes was reduced in the AAP + BET group compared with the AAP group (Fig. S8d), whereas autophagy-related markers, including LC3B and p62, were increased (Fig. S8e, f). Consistently, nuclear Nrf2 levels were elevated (Fig. S8g), and the Nrf2 target protein GSS was upregulated, whereas GCLM and GCLC remained unchanged (Fig. S8h). Together with the main pathological and biochemical findings, these data provide additional support for the association between betaine-mediated hepatoprotection and activation of the Keap1-Nrf2 pathway. Pearson correlation analyses further supported an association between Keap1 abundance and injury-related readouts, showing positive correlations between Keap1 and NAPQI levels (R = 0.8238; Fig. 3k) and between Keap1 and MPO-positive cells (R = 0.8727; Fig. S8i). Together, these results indicate that betaine mitigates AAP-induced acute liver injury and inflammation and is accompanied by reduced Keap1 levels, increased autophagy markers, and enhanced Nrf2 signaling consistent with improved GSH-dependent redox homeostasis.

### Betaine reduces liver injury, inflammation, and lipogenic signaling in MCD-induced MASLD

**Fig. 4:**
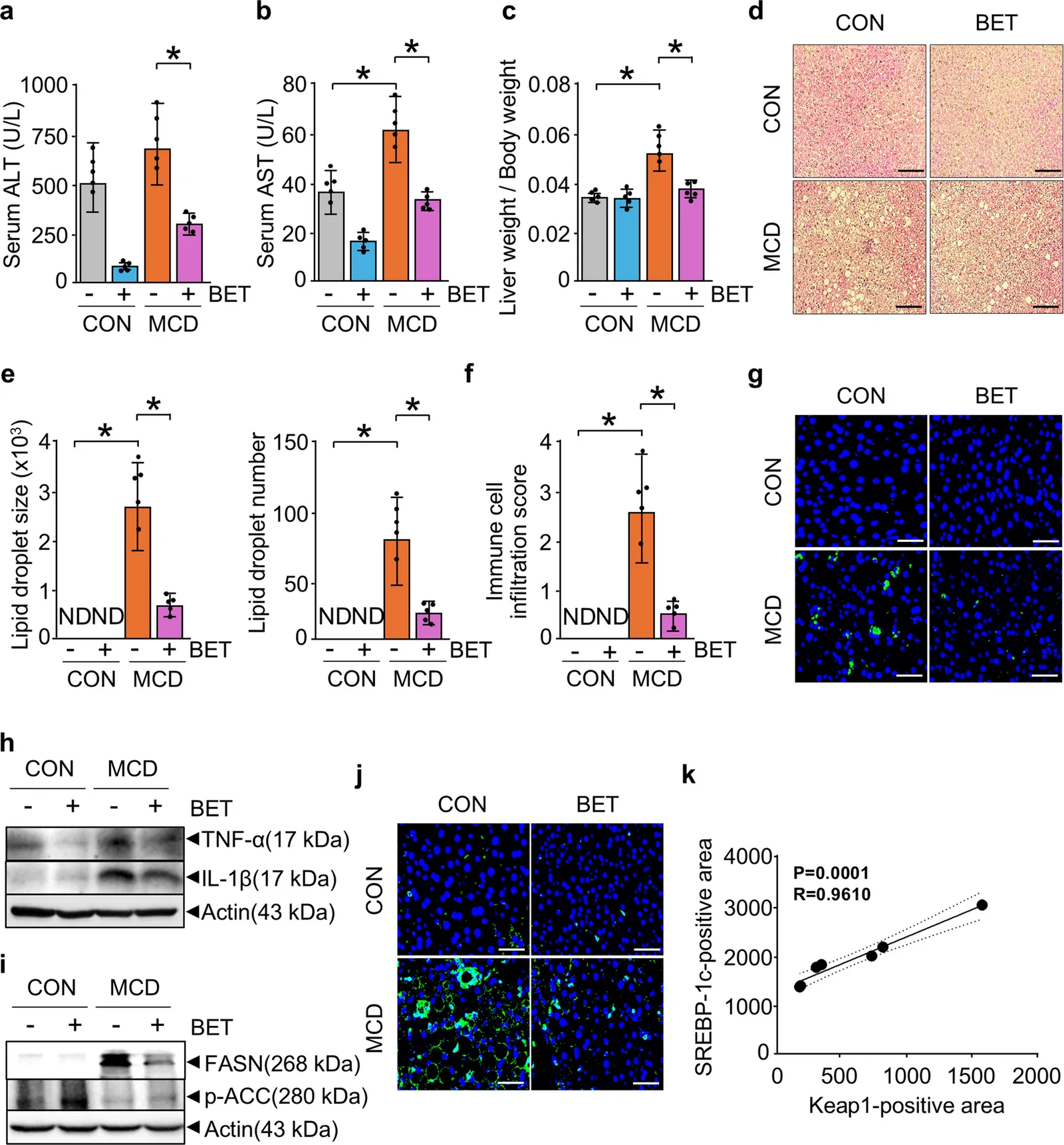
Betaine attenuates MCD diet–induced MASLD-like liver injury and lipogenic signaling. Seven-week-old male C57BL/6 mice were assigned to CON, BET, MCD, and MCD + BET groups. Betaine was administered by oral gavage at 40 mg/kg/day for 1 week before MCD feeding and continued during the 4-week diet period. Quantitative and representative image panels are presented using a consistent group order where applicable: CON, BET, MCD, and MCD + BET. **a**, **b** Serum ALT and AST levels. **c** Liver weight. **d** Representative H&E-stained liver sections for each group (200 ×; scale bar, 100 μm). **e**, **f** Histological scoring of steatosis/injury and inflammation based on H&E sections. ND, not determined. **g** Representative immunofluorescence images of MPO (green) and nuclei counterstained with DAPI (blue) in liver Sections. (400 ×; scale bar, 50 μm). **h** Hepatic inflammatory markers TNF-α and IL-1β. **i** Densitometric analysis of lipogenic proteins FASN and p-ACC. **j** Representative immunofluorescence images of SREBP-1c (green) and nuclei counterstained with DAPI (blue) in liver Sections. (400 ×; scale bar, 50 μm). **k** Pearson correlation between Keap1 and SREBP-1c levels (*n* = 7). Fluorescence channel identities are indicated in each panel. Data are presented as mean ± SD with individual values shown; *n* = 5 mice per group for a–j unless otherwise stated; *P* < 0.05

To evaluate the effects of betaine in an MCD diet–induced mouse model of MASLD, we assessed liver injury, histopathology, inflammation, and lipogenic signaling. Serum ALT and AST levels were significantly lower in the betaine-treated group than in the vehicle-treated MCD group (Fig. 4a, b), and the MCD-induced increase in liver weight was attenuated by betaine (Fig. 4c). H&E staining showed marked macrovesicular steatosis with enlarged lipid droplets and inflammatory cell infiltration in MCD-fed livers, whereas these histopathological changes were reduced by betaine (Fig. 4d–f). Betaine also decreased hepatic inflammation, as indicated by reduced MPO-positive cells and lower TNF-α and IL-1β protein levels (Fig. 4g, h). In parallel, betaine suppressed MCD-induced upregulation of lipogenic proteins, including Fatty acid synthase (FASN), Phospho-acetyl-CoA carboxylase (p-ACC), and Sterol regulatory element-binding protein 1c (SREBP-1c) (Fig. 4i, j). Immunofluorescence analysis of SREBP-1c (Fig. 4j) and Keap1 (Fig. S9) revealed parallel changes in their abundance, and Pearson correlation analysis showed a strong positive association between the two (R = 0.9610; Fig. 4k). These supplementary analyses further support the association between Keap1 abundance and injury- or lipogenesis-related readouts across liver injury contexts. Collectively, these results support a link between Keap1 levels and lipogenic activation in MCD-induced MASLD and show that betaine mitigates both hepatic injury and lipogenesis.

### RNA sequencing analysis of a MASLD cohort reveals transcriptomic associations with Nrf2-related redox pathways

To assess whether Nrf2-related redox pathway alterations are associated with human MASLD severity, publicly available RNA-seq data from liver tissues of 271 MASLD patients were obtained from the GEO database (GSE192959) and analyzed. Dysregulated GSH homeostasis and excessive oxidative stress have been implicated as important pathophysiological features of MASLD progression [15], providing a compelling rationale for investigating the directionality of gene expression regulated by *NFE2L2* (encoding Nrf2), the human transcript of Nrf2, in the MASLD cohort. Specifically, we examined disrupted expression patterns of Nrf2 target genes involved in intracellular redox homeostasis, along with the expression and correlation of *NFE2L2*, supporting the potential relevance of Nrf2-related redox pathways in the context of betaine-associated mechanisms in MASLD.

**Fig. 5:**
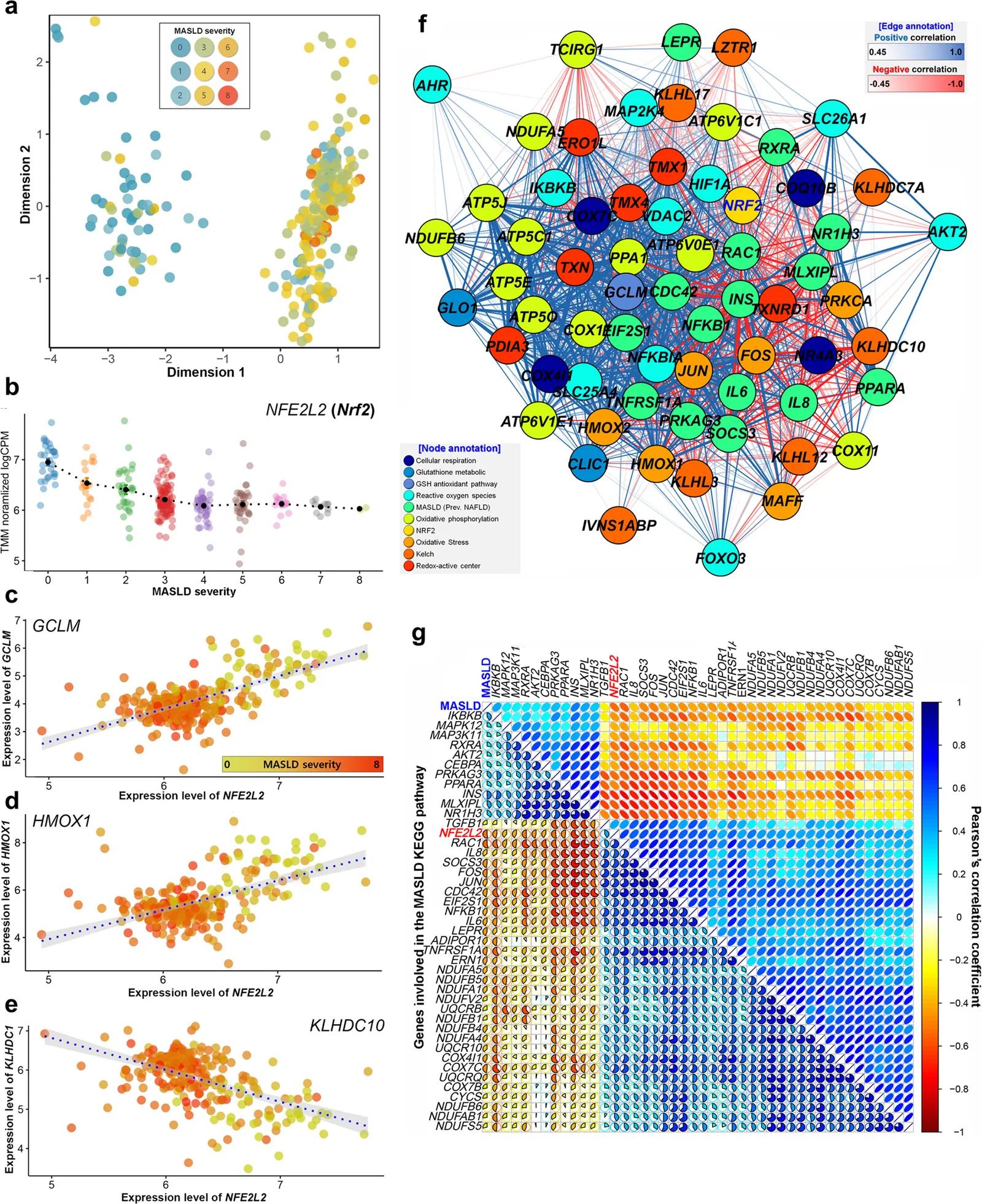
RNA sequencing analysis of a human MASLD cohort reveals transcriptomic associations with Nrf2-related redox pathways. **a** Dimensionality reduction plot for RNA-sequencing data generated from liver tissue of a total of 271 MASLD patients. Colors represent each MASLD activity score, with red indicating more severe levels. **b** Association between the expression level of Nrf2 transcript, *NFE2L2*, and MASLD activity score. **c** Co-expression pattern between *GCLM* and *NFE2L2*. **d** Co-expression pattern between *HMOX1* and *NFE2L2*. **e** Co-expression pattern between *KLHDC10* and *NFE2L2*. **f** Correlation-based network analysis results for co-expression patterns of genes significantly associated with MASLD activity score and functionally related pathways. **g** Co-expression patterns of gene clusters with two largely different directions based on *NFE2L2* and MASLD activity scores. The color scale represents row-scaled expression z-scores of the indicated genes, with lower and higher relative expression levels shown according to the accompanying color bar

Of particular interest, this analysis revealed a significant change in global gene expression patterns when the MASLD activity score (MAS) was 3 or higher (Fig. 5a). Notably, the expression level of *NFE2L2* (Padj: 9.02e-09) decreased linearly until the MAS reached 3, after which it plateaued (Fig. 5b). In addition, a strong positive correlation was observed between genes involved in GSH synthesis and the expression of *NFE2L2*, with a significant association with MAS (Fig. 5c, d). For example, *GCLM* exhibited a correlation of 0.635 with *NFE2L2* and decreased significantly as the MAS increased (Padj: 1.48e-09). Similarly, *HMOX1* showed a correlation of 0.545 with *NFE2L2* and was significantly associated with MAS (Padj: 2.46e-05). However, the *KEAP1* transcript did not show a significant association with *NFE2L2* or MAS (Fig. S10a). Of note, while no significant correlation was observed with *KEAP1*, various transcripts encoding proteins containing the Kelch domain negatively correlated with *NFE2L2* expression and were significantly associated with MAS (Fig. 5e and S10b).

Gene-set enrichment analysis (GSEA) was further performed for genes associated with MAS, identifying eight significant gene sets at a 5% significance level after false discovery rate (FDR) adjustment (Table S1). The Kelch domain gene set (Padj: 2.07e-07) was significantly enriched, comprising eight genes containing the Kelch motif. Pathways such as Redox Active Center (Padj: 2.80e-15), Glutathione Metabolic Process (Padj: 0.0008), and MASLD (Padj: 2.50e-47) were also significantly enriched. To further explore the role of Nrf2 target genes, we constructed a network based on their expression patterns, identifying 138 out of 170 genes with strong positive correlations (Fig. 5f). Notably, the majority of the genes in the well-established MASLD KEGG pathway were part of this network, supporting the robustness of the analysis (Fig. S10c). These 42 genes exhibited distinct expression patterns, forming two clusters that further reinforced the inverse association of *NFE2L2* and its GSH redox-associated target genes with MASLD progression (Fig. 5g). Additional supplementary analyses further supported this transcriptomic interpretation by showing the lack of a significant association for the *KEAP1* transcript, the involvement of Kelch-domain-containing transcripts, and the representation of MASLD KEGG pathway genes within the Nrf2-related network (Fig. S10a–c). Collectively, these findings provide transcriptomic evidence that Nrf2-related redox pathways are associated with MASLD severity, supporting a possible link between Nrf2 pathway suppression and disease progression rather than establishing direct therapeutic validation of betaine in human MASLD.

### Betaine-related genes and Nrf2 pathway signatures in a human MASLD RNA-seq cohort: transcriptomic support for a possible association

Analysis of the same GEO-derived human MASLD RNA-seq dataset revealed an inverse association between Nrf2 target genes involved in GSH redox homeostasis and MASLD severity, suggesting that repression of Nrf2-related redox programs may accompany MASLD progression. However, because this analysis was based on cross-sectional transcriptomic data, it could not determine whether betaine availability or betaine-related metabolism causally regulates Nrf2 activity in human MASLD. Based on the same GEO-derived human MASLD RNA-seq dataset (GSE192959), genes associated with betaine transport and metabolism were further examined to explore whether their expression patterns were aligned with *NFE2L2* expression and MASLD severity. These analyses were intended to provide additional correlative context rather than direct therapeutic validation of betaine supplementation.

**Fig. 6:**
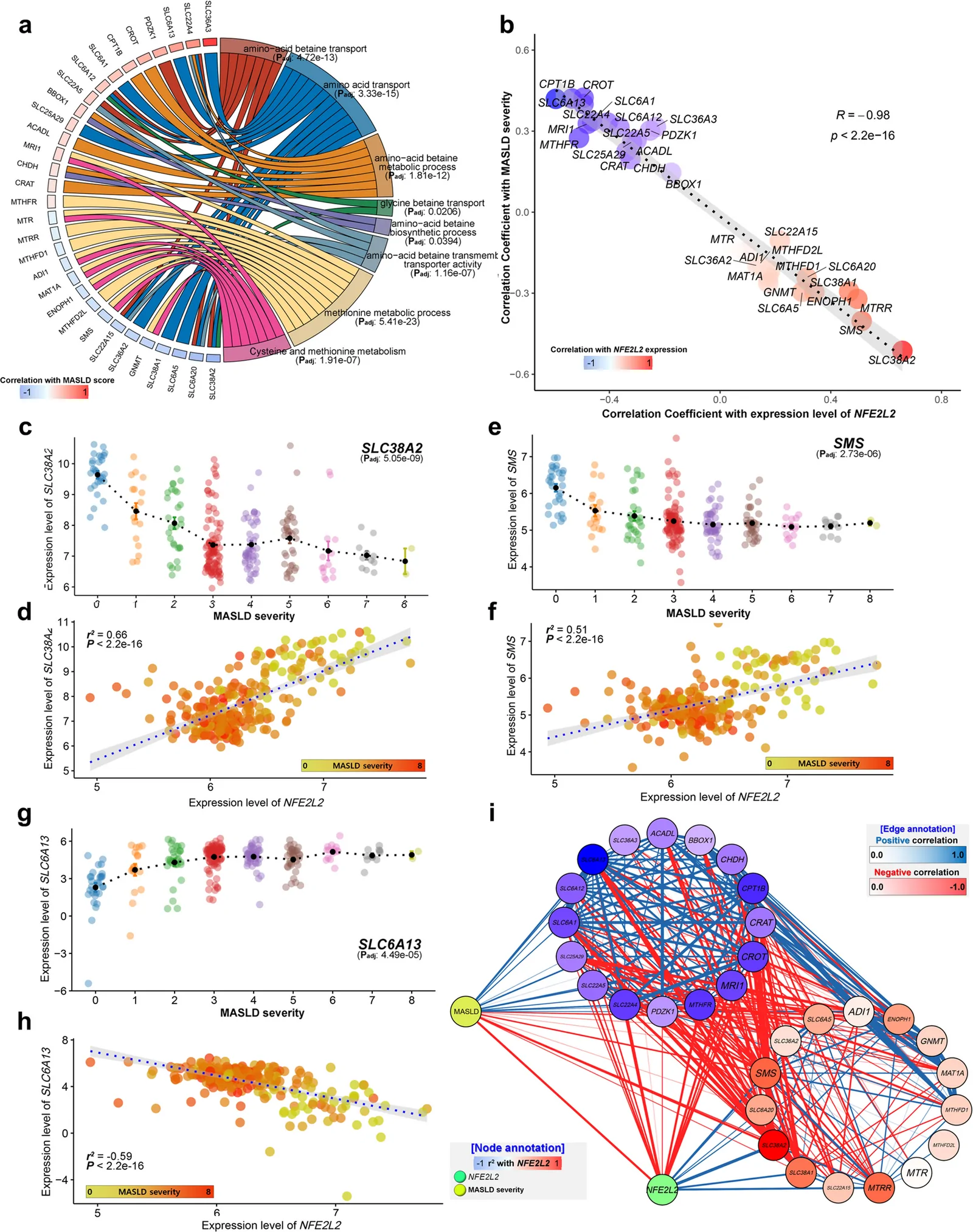
Human MASLD cohort analysis identifies coordinated changes in Nrf2 signaling and betaine-related genes. **a** Correlation among 31 genes involved in betaine-related pathways and MASLD activity score. Multiple ribbon colors are used only as categorical visual identifiers to distinguish enriched biological processes and their corresponding gene links, not as a rainbow-based continuous quantitative scale. Quantitative correlation information is shown separately using the blue-to-red color scale. **b** The 31 genes included in the betaine-related pathway have a strong association relationship in the opposite direction in correlation with the expression pattern of the Nrf2 transcript and MASLD activity score. **c** Association between *SLC38A2* expression level and MASLD activity score. **d** Co-expression pattern of *SLC38A2* with *NFE2L2*, a transcript of Nrf2. **e** Association between *SMS* expression level and MASLD activity score. **f** Co-expression pattern of *SMS* with *NFE2L2*. **g** Association between *SLC6A13* expression level and MASLD activity score. **h** Co-expression pattern of *SLC6A13* with *NFE2L2*. **i** Network plot of the two different directional associations between genes included in the betaine-related pathway, MASLD severity, and Nrf2

To address these questions, QuickGO [16] was used to identify 31 genes known to be involved in betaine metabolism that were significantly correlated with MAS (Fig. 6a; Table S2). Among these, 15 genes were negatively correlated with MAS, while the remaining 16 genes showed a positive correlation (Table S3). Genes positively correlated with MAS were particularly enriched in the amino acid betaine metabolic process (P_adj_: 1.81e-12), whereas those negatively correlated were enriched in the methionine metabolic process (P_adj_: 5.41e-23). For these 31 identified genes, their co-expression patterns with both MAS and *NFE2L2* were further examined, revealing a marked linear relationship (Fig. 6b). These findings suggest a correlative relationship among betaine-related genes, *NFE2L2* expression, and MAS, but do not establish whether altered betaine metabolism directly contributes to Nrf2 pathway suppression or MASLD progression. To further elucidate the mechanistic link, the functional roles of the identified genes were analyzed in relation to betaine physiology. First, *SLC38A2* (encoding Sodium-coupled neutral amino acid symporter 2), which showed a strong positive correlation with *NFE2L2* and was downregulated in severe MASLD (Fig. 6c, d), functions as a sodium-dependent amino acid transporter capable of mediating betaine uptake into hepatocytes, particularly under stress conditions [17]. Its downregulation suggests a compromised capacity for cellular betaine uptake in advanced MASLD. Similarly, *SMS* (encoding Spermine synthase), also positively correlated with *NFE2L2* (Fig. 6e, f), is a key enzyme in polyamine biosynthesis. This process is tightly coupled to the methionine cycle, where betaine serves as a crucial methyl donor to regenerate methionine and maintain the S-adenosylmethionine (*SAM*) pool required for polyamine synthesis [18]. The reduced expression of *SMS* implies a disruption in the downstream metabolic flux dependent on methyl donors. In contrast, *SLC6A13*, known to transport both GABA and betaine with high affinity [19], was upregulated and negatively correlated with *NFE2L2* (Fig. 6g, h). This inverse pattern may represent a compensatory but insufficient response to the cellular stress or an alteration in transport specificity under pathological conditions. More importantly, these genes exhibit consistent directional effects on MASLD progression (Fig. 6i). Collectively, the expression patterns of these transporter and metabolic genes suggest a possible association between betaine-related transport/metabolic networks and MASLD severity, in parallel with repression of the Nrf2 antioxidant system. However, because these analyses are based on transcriptomic correlations, they should be interpreted as hypothesis-generating rather than as direct functional validation of betaine utilization in human MASLD.

## Discussion

Toxicants and ROS can induce chemical and oxidative stress in cells and organisms, requiring coordinated detoxifying and antioxidant responses to maintain redox homeostasis [1, 2]. The Keap1–Nrf2 signaling pathway is widely recognized as an important component of this protective response and has been explored as a therapeutic target in redox-associated diseases [7, 20]. Several natural electrophilic compounds have been reported to modulate Keap1–Nrf2 signaling, although the precise mechanisms can vary depending on the compound and cellular context [20]. However, previous studies have struggled to establish a direct interaction between test compounds and Keap1. This study aimed to investigate the role of betaine in regulating Keap1 as a trigger for Nrf2 signaling and to elucidate the underlying molecular mechanism involved.

Betaine is a nutrient present in nearly all cell types, with the liver requiring it primarily as a methyl donor [21, 22]. Given the liver’s central role in producing various biochemical reactions that lead to cellular stress, the active function of Keap1 as a stress sensor is particularly critical in this organ [23, 24]. Consequently, the liver was selected as the model system to investigate regulation of Keap1-Nrf2 by betaine. Betaine has generally been reported to have a favorable safety profile in previous evaluations [25], which is consistent with the lack of detectable cytotoxicity observed in AML12 cells under the conditions used in this study. MTT and LDH assays showed no toxicity in AML12 cells after 24 h of treatment with 100 mM betaine (data not shown). However, a concentration of 100 μM was chosen for subsequent experiments, aligning with the physiological concentration of betaine in human blood (approximately 50 μM) while accounting for the anti-cellular uptake factor [26, 27].

Under homeostatic conditions, Nrf2 is ubiquitinated by the Keap1-Cul3-E3 ubiquitin ligase complex and degraded by the 26S proteasome [28]. During electrophilic stress, however, modifications of Keap1 inhibit E3 ubiquitin ligase activity, leading to stabilization of Nrf2 [29]. Keap1 can be modified either through conformational changes (canonical pathway) or via p62-mediated autophagic degradation (non-canonical pathway) [28]. The present findings are consistent with the possibility that betaine preferentially engages a p62-associated autophagic mechanism to regulate the Keap1-Nrf2 pathway. This interpretation is supported by the reversal of betaine-induced Keap1 reduction upon CQ treatment. Furthermore, betaine did not affect Keap1 mRNA levels, suggesting that transcriptional suppression is unlikely to be the major mechanism underlying the observed reduction in Keap1 protein levels. These findings align with recent evidence highlighting the role of autophagy in Keap1 degradation [3, 30]. Additionally, when betaine was administered together with the proteasome inhibitor MG132, no change in the expression of the Nrf2 target gene Gss was observed (Fig. S11), further supporting this pathway.

Although betaine has been shown to enhance Nrf2 activity and promote the expression of its target genes in various disease models [14, 31, 32], the mechanism underlying Nrf2 activation remained unclear. Keap1 contains abundant cysteine residues that serve as sensors and can be modified by electrophilic molecules under environmental stress or in response to synthetic drugs and natural products [3, 33–35]. Ghyczy et al. hypothesized that natural products containing electrophilic methyl groups, including betaine, mitigate disruptions in redox homeostasis [36]. Based on this, we initially hypothesized that betaine directly binds to cysteine residues to modify Keap1. However, the present data suggest that betaine-associated methylation occurs at Cys513 and Cys518 of Keap1 rather than through stable binding of intact betaine to the protein. Betaine is known to transfer methyl groups to target molecules via the enzymatic activity of betaine-homocysteine S-methyltransferase [9]. However, our findings suggest that this methyl transfer occurs through non-enzymatic reactions involving structural features of Keap1, as demonstrated by LC–MS analysis of purified Keap1 protein incubated with betaine. Further investigations are required to elucidate the precise mechanism of methyl group transfer by betaine.

The Kelch domain of Keap1, containing Cys513 and Cys518, mediates protein–protein interactions and serves as a binding domain for both p62 and Nrf2 [37]. Keap1 comprises three domains: BTB, IVR, and Kelch. Variants in the BTB and IVR domains have been associated with altered interactions between Keap1 and the CUL3 protein, leading to the release of Nrf2 [38]. The long loop connecting the IVR and Kelch domains, coupled with the occurrence of betaine-induced methylation within the Kelch domain, suggests that this modification is unlikely to directly affect its interaction with CUL3. Instead, we propose that methylation modifies the conformation of the Kelch domain's binding site for Nrf2 and p62. Our findings suggest that this conformational change may enhance the domain's binding affinity for p62. However, because the present study primarily focused on the p62-mediated non-canonical pathway of Nrf2 activation, we did not directly investigate whether betaine-induced methylation of Keap1 also alters the Keap1-Nrf2 interaction itself. Therefore, we cannot exclude the possibility that methylation at Cys513/518 may additionally influence Nrf2 binding dynamics. Further structural and biochemical studies will be required to determine how Keap1 methylation affects the relative interactions of Keap1 with both p62 and Nrf2.

Collectively, these mechanistic findings are consistent with improvements in hepatic GSH availability and GSH-dependent redox homeostasis, supporting further evaluation of betaine as a potential strategy for liver diseases characterized by redox dyshomeostasis, including AAP-induced acute liver injury/failure and MASLD. Although the AAP model primarily reflects acute GSH depletion, activation of the Keap1–Nrf2 pathway constitutes a conserved cytoprotective program that may also be relevant in other oxidative stress-associated liver injury contexts. However, because the present mechanistic analyses were primarily performed in the AAP model, broader applicability of the proposed mechanism to other forms of liver injury remains to be experimentally established. Consistent with this possibility, previous studies have reported protective effects of betaine in carbon tetrachloride (CCl₄)- and ethanol-induced models [39, 40]. In parallel, our human MASLD transcriptomic analysis provides correlative and hypothesis-generating context by showing repression of the *NFE2L2* (Nrf2) program alongside coordinated shifts in betaine-related genes. In particular, genes positively correlated with *NFE2L2*—including the betaine-associated transporter *SLC38A2* and the polyamine enzyme *SMS*—were reduced in severe MASLD, whereas *SLC6A13*, which was inversely correlated with *NFE2L2*, increased with disease severity (Fig. 6c–h). Together, these observations are consistent with the idea that disease progression is accompanied by attenuation of Nrf2-aligned redox defenses and remodeling of betaine transport/metabolic programs, supporting the rationale for evaluating betaine supplementation as a means to restore Nrf2-mediated redox capacity in MASLD. However, because the present mechanistic analyses were primarily performed in the AAP-induced acute liver injury model, further validation in additional oxidative stress–associated liver injury models will be required to determine the broader generalizability of the proposed mechanism.

In this context, several limitations should be considered when interpreting these findings. First, although betaine-associated methylation of recombinant Keap1 was detected in vitro and the functional requirement of Cys513/Cys518 was supported using site-directed mutants in cells, the biochemical mechanism by which betaine mediates methylation at these residues remains incompletely defined. One plausible model is that methyl transfer may occur through a non-enzymatic process facilitated by the local microenvironment of the Kelch domain. Specifically, microenvironmental effects may lower the pKa of Cys513 and Cys518, increasing the fraction of thiolate anions at physiological pH; these nucleophilic thiolates could, in principle, promote methyl transfer from a quaternary ammonium donor such as betaine via an SN2-like reaction [41–43]. Consistent with this possibility, previous mass spectrometry–based analyses have identified Cys513 and Cys518 within the Keap1 Kelch domain as reactive cysteine residues susceptible to covalent modification [44]. Nevertheless, enzyme-dependent contributions cannot be excluded. For example, betaine-dependent one-carbon metabolism may alter methyl-donor availability and thereby indirectly influence cysteine methylation dynamics. Future studies will be required to determine the relative contributions of SAM-dependent methylation pathways and non-enzymatic chemistry in establishing Keap1 methylation at Cys513/Cys518. In addition, although the present data support enhanced p62-associated autophagic degradation of Keap1, whether Keap1 methylation also alters Keap1–Nrf2 binding dynamics remains to be determined. Second, Keap1 methylation at Cys513 and Cys518 was not directly detected in mouse liver tissues. One possible explanation is that methylation triggers rapid p62-mediated autophagic degradation of Keap1, limiting the steady-state abundance of the modified species below the detection threshold. Alternatively, Keap1 methylation may be transient and counteracted by currently undefined demethylation or repair mechanisms in vivo. Additional studies with more sensitive detection methods will be needed to clarify whether and how Keap1 methylation occurs and turns over in physiological liver tissue. Third, the human MASLD cohort analysis was based on publicly available liver RNA-seq datasets, which precluded measurement of concurrent plasma betaine levels. Thus, repression of the *NFE2L2* pathway could not be directly correlated with endogenous betaine status in the same individuals. In addition, this analysis was not independently validated by qPCR, protein-level measurements, or experimental manipulation in an animal model. Therefore, the human RNA-seq data should be interpreted as correlative transcriptomic support for a possible association between betaine-related transport/metabolic networks, Nrf2 pathway activity, and MASLD progression, rather than as direct translational validation. Nonetheless, prior clinical evidence reporting betaine insufficiency in steatohepatitis [45] and potential improvement of hepatic steatosis after betaine supplementation [46] provides useful clinical context for the mechanistic findings presented here.

## Conclusion

Overall, this study provides evidence that betaine may enhance Nrf2 target gene expression through modulation of Keap1. Our findings support Keap1 Cys513/Cys518 methylation as a potential post-translational regulatory event associated with p62-mediated autophagic degradation of Keap1, Nrf2 stabilization, and reinforcement of GSH-dependent redox homeostasis. Across biochemical assays, in silico modeling, in vitro studies, and in vivo liver injury models, these data provide mechanistic support for further evaluating betaine-related Keap1–Nrf2 regulation in MASLD. However, additional studies using direct in vivo detection of Keap1 methylation, genetic pathway validation, and prospective human or clinically relevant models will be required before establishing the translational significance of this mechanism.

## Materials and methods

Detailed experimental procedures are provided in the Supplementary Methods. Briefly, essential information required for reproducibility is described below; extended protocols (including Keap1 mutant construction, LC–MS workflow details, molecular dynamics simulations, PET–CT processing, and the full RNA-seq/bioinformatic pipeline) are described in the Supplementary Methods. Antibody information is provided in Table S4.

### Cell culture and treatments

AML12 mouse hepatocytes (ATCC) were cultured in DMEM/F12 supplemented with 10% fetal bovine serum, penicillin–streptomycin (100 U/mL), ITS (5 μg/mL), and dexamethasone (0.1 mM) at 37 °C with 5% CO₂. Cells (passages 15–20) were seeded at 5 × 10^4^ cells/cm^2^ and treated with betaine (0–100 μM) for 3 h unless otherwise indicated, followed by RNA or protein extraction.

### Animal studies and experimental design

All animal experiments were approved by the Korea University IACUC (KUIACUC-2023-0014). Male C57BL/6 mice were maintained under standard conditions with ad libitum access to food and water. For the AAP model, eight-week-old mice were assigned to Control (CON), betaine (BET), AAP, and AAP plus betaine (AAP + BET) groups. Betaine (40 mg/kg/day, oral gavage) was administered for 7 days, and AAP (500 mg/kg, oral) was given 2 h after the final betaine dose. Mice were sacrificed 12 h after AAP administration for blood and liver collection.

For the MCD diet model, seven-week-old male C57BL/6 mice were acclimatized for 1 week and randomized into Control (CON), betaine (BET), MCD, and MCD plus betaine (MCD + BET) groups (*n* = 8 per group). Betaine (40 mg/kg/day, dissolved in PBS) was administered by oral gavage for 1 week prior to MCD feeding and continued during the diet period. Mice were fed an MCD diet for 4 weeks to induce steatohepatitis-like pathology, and tissues and serum were collected at the endpoint for biochemical and histological analyses. Additional procedural details are provided in the Supplementary Methods.

### Immunoblotting and co-IP

Liver tissues were lysed in ice-cold RIPA buffer containing protease inhibitors and PMSF. After homogenization and centrifugation, protein concentrations were determined by BCA assay, and equal amounts were resolved by SDS–PAGE and transferred to NC or PVDF membranes. Membranes were blocked and incubated with primary antibodies (Table S4), followed by HRP-conjugated secondary antibodies and chemiluminescent detection. For co-IP, clarified lysates were incubated with antibody-bound magnetic Protein A/G beads at 4 °C overnight, washed, and eluted for immunoblotting.

### RNA isolation and quantitative real-time PCR

Total RNA was extracted using TRIzol, and 1 μg RNA was reverse-transcribed to cDNA. Quantitative PCR was performed using GoTaq qPCR Master Mix on a QuantStudio 3 system. Relative expression was calculated by the ΔΔCt method with GAPDH as the reference gene. Primer sequences are listed in Table S5.

### Immunocytochemistry and tissue immunofluorescence

For immunocytochemistry, AML12 cells were fixed with cold methanol, permeabilized, blocked, and incubated with primary antibodies overnight at 4 °C, followed by fluorescent secondary antibodies and DAPI mounting. For tissue immunofluorescence, liver samples were paraffin-embedded, sectioned (3 μm), rehydrated, subjected to antigen retrieval, blocked, and stained with primary and Alexa Fluor–conjugated secondary antibodies. Images were acquired using a Zeiss Axio microscope and analyzed with ImageJ.

### Nuclear fractionation

Nuclear extracts from liver tissue were prepared using the NE-PER kit according to the manufacturer’s protocol, followed by immunoblotting to assess nuclear Nrf2 levels.

### Serum ALT/AST and histology

Serum ALT and AST levels were measured using commercial assay kits according to the manufacturers’ instructions. For histological evaluation, paraffin-embedded liver Sections. (3 μm) were stained with hematoxylin and eosin (H&E) using standard procedures.

### Human MASLD RNA-seq analysis

Publicly available RNA-seq data from a published human MASLD cohort were downloaded from the GEO database under accession number GSE192959 (*n* = 271) and analyzed after quality control. Differential association testing with MASLD activity score was performed using limma-voom with covariates including fibrosis stage, age, and sex, and significance was assessed after FDR correction. Functional enrichment and network analyses were conducted as described in the Supplementary Methods.

### Statistical analysis

Data are presented as mean ± SD. Statistical analyses were performed using GraphPad Prism 9. Two-group comparisons were conducted using an unpaired (two-tailed) Student’s t-test unless otherwise stated. A *P* value < 0.05 was considered statistically significant. Statistical significance is denoted as *P* < 0.05 (*), P < 0.01 (**), P < 0.001 (***), and *P* < 0.0001 (****).

## Supplementary Information

Supplementary Material 1.

## Data Availability

The authors declare that all data supporting the findings of this study are available within the paper, and additional data can be obtained by contacting the corresponding authors via email.
