# Supplementary material for: Betaine-driven Keap1 methylation promotes p62-mediated autophagy and Nrf2 stabilization to alleviate glutathione-dependent redox dyshomeostasis in liver injury
Source: Mol Biomed. 2026 Sep 16;7:172. doi: 10.1186/s43556-026-00579-1 (PMC13582688; doi:10.1186/s43556-026-00579-1)
Supplement: Supplementary file 1 [file 43556_2026_579_MOESM1_ESM.doc]

**Supplementary Information**

**Betaine-driven Keap1 methylation promotes p62-mediated autophagy and Nrf2 stabilization to alleviate glutathione-dependent redox dyshomeostasis in liver injury**

Moon Han Chang^1†^, Chae Hwan Lee^2,3,4†^, Young Rae Cho^4,5^, Ryeo Gang Son^4,5^, Seoyoon Lee^6^, Shinwoo Park^4,7^, Jaeryeong Kim^4,7^, Young Ho Koh^1^, Art E Cho^4,5^, Seung Pil Pack^4,5*^, Minseok Seo^4,7*^, Jin Hyup Lee^2,3,4,8*^, Hanvit Cha^2,4*^

^1^Division of Brain Disease Research, Department of Chronic Disease Convergence Research, Korea National Institute of Health, Cheongju, Republic of Korea, ^2^Department of Food and Biotechnology, Korea University, Sejong, Republic of Korea; ^3^BK21 FOUR Research Group for Omics-based Bio-health in Food Industry, Korea university, Sejong, Republic of Korea; ^4^Biological Clock-based Anti-aging Convergence RLRC, Korea university, Sejong, Republic of Korea; ^5^Department of Biotechnology and Bioinformatics, Korea University, Sejong, Republic of Korea; ^6^Advanced Drug Quality Division Department, National Institute of Food and Drug Safety Evaluation, Ministry of Food and Drug Safety, Cheongju, Republic of Korea; ^7^Department of Computer and Information Science, Korea University, Sejong, Republic of Korea; ^8^Institutes of Natural Sciences, Korea University, Sejong, Republic of Korea

^†^These authors contributed equally to this work.

^*^Correspondence: Seung Pil Pack (spack@korea.ac.kr), (Minseok Seo (mins@korea.ac.kr), Jin Hyup Lee (jinhyuplee@korea.ac.kr), and Hanvit Cha (chahv@korea.ac.kr).

**Supplementary Methods**

**Reagents and detailed treatment conditions**

Betaine, dimethylglycine, and acetaminophen (purity ≥98%) were purchased from Sigma-Aldrich (MO, USA). Chloroquine, MG132 (purity ≥98.5%), and HisPur™ Ni-NTA Resin were purchased from Thermo Fisher Scientific (MO, USA). Co-immunoprecipitation kits and magnetic bead–conjugated Protein A/G were obtained from Bio-Rad (MA, USA). Antibody information is provided in Supplementary Table 4. For cell-based assays, AML12 cells were treated with betaine at the indicated concentrations (0–100 μM) for 3 h unless otherwise specified. For inhibitor experiments, chloroquine (CQ) or MG132 was applied at concentrations and durations indicated in the corresponding figure legends. The primer sequences used for real-time qPCR analyses are provided in Supplementary Table 5.

**Acetaminophen (AAP)-induced acute liver injury model**

All animal procedures were approved by the Korea University IACUC (KUIACUC-2023-0014). Eight-week-old male mice were housed under a 12-h light/dark cycle with ad libitum access to food and water. After acclimatization, mice were randomized into four groups (Control, BET only, AAP only, and AAP + BET). Betaine (40 mg/kg) was administered by oral gavage once daily for 7 consecutive days, and acetaminophen (AAP; 500 mg/kg) was administered orally 2 h after the final betaine treatment. Mice were euthanized 12 h after AAP administration, and blood and liver tissues were collected for downstream analyses.

**Methionine- and choline- deficient (MCD) diet–induced steatotic liver disease model**

Seven-week-old male C57BL/6 mice were purchased and acclimatized for 1 week before experiments. Mice were randomized into four groups (Control, BET, MCD, and MCD + BET; n = 8 per group). Betaine was dissolved in PBS and administered by oral gavage (40 mg/kg/day) as a pretreatment for 1 week prior to MCD feeding, and administration was continued throughout the diet period. To induce steatohepatitis-like pathology, mice were fed a MCD diet for 4 weeks, while control groups received the corresponding control diet. At the endpoint, mice were euthanized and blood and liver tissues were collected. Liver weight was recorded, and samples were processed for serum ALT/AST assays, H&E staining for histopathology, immunofluorescence staining (including MPO) to assess inflammatory cell infiltration, and immunoblotting for inflammatory cytokines (e.g., TNF-α and IL-1β) and lipogenic markers (FASN, p-ACC, and SREBP-1c).

**Keap1 mutant plasmid construction**

To investigate Keap1–betaine interaction and identify functional cysteine residues, the Keap1 template was derived from pcDNA3-HA2-Keap1 (Addgene #21556; gift from Yue Xiong). The vector was modified to generate pcDNA3-His-HA-Keap1 by adding a Kozak sequence, incorporating a His tag for immunoprecipitation, and optimizing the GC ratio around target cysteine residues. Using primers P1 and P2, a first PCR fragment containing the HA tag and partial Keap1 sequence was generated (Supplementary Table 6). This fragment was used as a template for a second PCR to introduce the His tag and Keap1 mutation variants (WT, C513A, C518A, C513A/C518A) using forward primer P3 and reverse primers P4–P7. The pcDNA-Kozak backbone was obtained by inverse PCR using primers P8 and P9. PCR products were assembled into pcDNA3-His-HA-Keap1 constructs using an Overlap Cloner Kit (ELPIS Biotech, Daejeon, South Korea) following a ligation-independent cloning method (Supplementary Table 7). Constructs were transformed into *E. coli* DH5α (RBC, Taipei, Taiwan) and propagated in Luria broth containing ampicillin (100 μg/mL). Plasmids were purified for downstream transfection experiments. Primer sequences for Keap1 mutations are listed in Supplementary Table 8.

**Keap1–betaine thermal shift assay**

Protein thermal stability was assessed using the GloMelt™ Thermal Shift Protein Stability Kit (Biotium, CA, USA) according to the manufacturer’s protocol. Human recombinant Keap1 protein (0.06 μM) was prepared in the presence or absence of betaine (1.2 μM). Keap1 and GloMelt dye were mixed in PBS and loaded into a qPCR instrument for melt analysis. The qPCR program included an initial hold at 25°C for 30 s, followed by a melt/dissociation phase from 25°C to 99°C at a ramp rate of 0.05°C/s. Melting profiles were compared between groups.

**LC–MS analysis for Keap1 methylation**

For methylation analysis, human recombinant Keap1 protein was initially supplied in a buffer containing 10% glycerol, 500 mM NaCl, and 3 mM DTT; therefore, buffer exchange was performed prior to reaction. The buffer was exchanged into recombinant protein storage buffer (50 mM Tris-HCl, 10 mM reduced glutathione, pH 8.0) and adjusted to 4 μg/40 μL. Keap1 was incubated with betaine (1.2 μM) for 2 h, and the reaction was quenched with DTT (final 60 μM). Samples (1 μg/10 μL) were denatured at 95°C for 10 min, resolved by SDS-PAGE, and subjected to in-gel trypsin digestion at 37°C for 16 h. Digested peptides were dried, reconstituted in 0.1% formic acid, and analyzed by LC–MS at the Korea Basic Science Institute. LC–MS data were used to identify methylated residues in Keap1.

**Molecular docking and molecular dynamics (MD) simulations**

**Protein preparation**

The Keap1 Kelch domain structure was prepared based on PDB ID 3WDZ, which includes a p62-binding interface. Protein preparation was performed using Maestro/Schrödinger. Methylation modifications at Cys513 and Cys518 were introduced to generate the methylated Keap1 model in addition to the wild-type structure. Protonation states at pH 7.4 were assigned using Epik. Both structures were minimized using the OPLS4 force field in the Prime module.

**MD simulation**

MD simulations were performed using the Desmond module. Systems were placed in a cubic water box with a 15 Å buffer from the protein surface and solvated using the TIP3P water model. Na^+^ and Cl^-^ ions were added to neutralize the system and achieve an ionic strength of 0.15 M. After equilibration using the Desmond Relax protocol, temperature control was performed using a Nose–Hoover chain thermostat under the NVT ensemble. Heating and cooling cycles were run from 0 K to 800 K over 10 ps and cooled to 300 K over 10 ps, repeated for 20 cycles, to sample stable conformations. The most stable structure was selected using principal component analysis (PCA) and free energy landscape (FEL) analysis, followed by additional equilibration. Production MD was performed at 300 K and 1 bar using the Nose–Hoover thermostat and the Martyna–Tobias–Klein barostat for 30 ns.

**MD trajectory analysis**

Trajectory analyses were performed to compare wild-type and methylated Keap1 systems, including PCA- and FEL-based evaluation to identify stable conformational states. Additional analysis parameters and scripts were applied as implemented in the Schrödinger suite.

**Micro–CT imaging acquisition and image processing**

To evaluate pathological changes in the liver, mice treated with betaine and/or AAP were injected with the contrast agent IR-117. After euthanasia using ketamine, animals were scanned using a micro-PET/CT system at the Korea Basic Science Institute. Image processing, grayscale presentation, and quantitative evaluation were performed using ImageJ. Acquisition and reconstruction parameters were applied according to institutional core facility settings.

**Expanded bioinformatic workflow for human MASLD RNA-seq**

A total of 271 RNA-seq datasets derived from liver tissue of a published MASLD cohort (GSE192959) were analyzed. After quality control, statistical modeling was performed using limma-voom based on the count matrix pre-processed in the original study. MASLD activity score, fibrosis stage, age, and sex were included as covariates in the linear model. For association tests with MASLD activity score, hypothesis testing was conducted using eBayes, and statistical significance was assessed at a 5% significance level after false discovery rate (FDR) correction. Functional enrichment analyses were performed using DAVID, and terms with FDR-adjusted P < 0.05 were considered significant. QuickGO was used to identify genes involved in betaine-related pathways. Network analysis was conducted using qgraph (v1.9.8) with a minimum correlation of 0.4, cut threshold of 0.6, and spring layout.

**Supplementary Table 1.** Significantly identified pathways enriched in genes that associated with NAFLD activity.

| Category | Term | # of Genes | % | p Value | FDR adjusted P |
| --- | --- | --- | --- | --- | --- |
| UP_KW_DOMAIN | KW-0676~Redox-active center | 13 | 11.711 | 1.75E-16 | 2.80E-15 |
| SMART | SM00612:Kelch | 8 | 7.207 | 5.31E-09 | 2.07E-07 |
| GOTERM_BP_DIRECT | GO:0045333~cellular respiration | 11 | 9.909 | 2.20E-14 | 8.97E-12 |
| GOTERM_BP_DIRECT | GO:0006749~glutathione metabolic process | 5 | 4.504 | 1.31E-04 | 0.008009312 |
| BIOCARTA | h_arenrf2Pathway:Oxidative Stress Induced Gene Expression Via Nrf2 | 8 | 7.207 | 1.63E-07 | 2.19E-05 |
| KEGG_PATHWAY | hsa00190:Oxidative phosphorylation | 31 | 27.927 | 2.40E-32 | 9.85E-31 |
| KEGG_PATHWAY | Non-alcoholic fatty liver disease | 42 | 38.900 | 2.20E-49 | 2.50E-47 |
| KEGG_PATHWAY | Chemical carcinogenesis - reactive oxygen species | 48 | 43.243 | 5.84E-51 | 7.19E-49 |

**Supplementary Table 2.** List of 31 genes involved in betaine-related pathways with significant association with NAFLD activity score.

| geneSymbol | Slope with NAFLD activity score | AvgExpr | P | adjP |
| --- | --- | --- | --- | --- |
| SLC38A2 | -0.234003819 | 7.750526 | 3.92E-11 | 5.05E-09 |
| SLC6A20 | -0.203929937 | -3.046 | 0.007067 | 0.017828 |
| SLC6A5 | -0.191011197 | -4.50705 | 0.000233 | 0.001088 |
| SLC38A1 | -0.142198592 | 4.422377 | 3.98E-05 | 0.000259 |
| GNMT | -0.134412202 | 6.165893 | 0.000163 | 0.000808 |
| SLC36A2 | -0.130238664 | -4.38985 | 0.01707 | 0.036814 |
| SLC22A15 | -0.129006808 | 1.114229 | 0.014835 | 0.032735 |
| SMS | -0.115378814 | 5.339296 | 1.42E-07 | 2.73E-06 |
| MTHFD2L | -0.094210515 | 3.473074 | 0.007756 | 0.019261 |
| ENOPH1 | -0.091162298 | 2.728235 | 0.000212 | 0.001006 |
| MAT1A | -0.084971364 | 9.938311 | 0.000647 | 0.002493 |
| ADI1 | -0.074885712 | 5.489464 | 0.006716 | 0.017073 |
| MTHFD1 | -0.053211735 | 9.909785 | 0.00444 | 0.012126 |
| MTRR | -0.046672499 | 5.944678 | 0.002321 | 0.007109 |
| MTR | -0.034399435 | 7.722281 | 0.008391 | 0.02055 |
| MTHFR | 0.051421468 | 4.824361 | 0.010378 | 0.024432 |
| CRAT | 0.064179298 | 5.673269 | 0.001025 | 0.003625 |
| CHDH | 0.064413431 | 3.834582 | 0.004884 | 0.013149 |
| MRI1 | 0.076082878 | 2.305733 | 0.000772 | 0.002881 |
| ACADL | 0.079706412 | 5.999411 | 0.000289 | 0.001293 |
| SLC25A29 | 0.086895128 | 3.680827 | 0.001679 | 0.00543 |
| BBOX1 | 0.088982125 | 4.348454 | 0.010941 | 0.025526 |
| SLC22A5 | 0.097737641 | 4.911985 | 0.00011 | 0.000587 |
| SLC6A12 | 0.11309718 | 5.806283 | 1.99E-05 | 0.000147 |
| SLC6A1 | 0.117387823 | 6.432469 | 1.13E-05 | 9.32E-05 |
| CPT1B | 0.124917903 | 5.787151 | 4.04E-08 | 1.01E-06 |
| CROT | 0.156462264 | 5.962159 | 4.13E-07 | 6.34E-06 |
| PDZK1 | 0.169233748 | 3.571204 | 3.94E-07 | 6.12E-06 |
| SLC6A13 | 0.200311349 | 4.371808 | 4.63E-06 | 4.49E-05 |
| SLC22A4 | 0.224371561 | 5.293317 | 1.46E-05 | 0.000114 |
| SLC36A3 | 0.361562234 | -3.70125 | 2.57E-07 | 4.35E-06 |

**Supplementary Table 3.** Genes belonging to pathways enriched with genes showing significant associated with NAFLD activity score.

| Enesembl_ID | geneSymbol | Gene_type | Slope | AvgExpr | P | adjP | signWithNrf2 | corWithNrf2 | GENE NAME | Pathway |
| --- | --- | --- | --- | --- | --- | --- | --- | --- | --- | --- |
| ENSG00000091140 | DLD | protein_coding | -0.07023 | 6.897051 | 7.8E-05 | 0.000444 | + | 0.437201152 | dihydrolipoamide dehydrogenase(DLD) | UP_KW_DOMAIN: Redox-active center |
| ENSG00000091164 | TXNL1 | protein_coding | -0.07292 | 4.979515 | 0.000935 | 0.003361 | + | 0.448945543 | thioredoxin like 1(TXNL1) | UP_KW_DOMAIN: Redox-active center |
| ENSG00000117592 | PRDX6 | protein_coding | -0.142 | 6.372893 | 2.42E-08 | 6.84E-07 | + | 0.434891289 | peroxiredoxin 6(PRDX6) | UP_KW_DOMAIN: Redox-active center |
| ENSG00000117862 | TXNDC12 | protein_coding | -0.0831 | 3.852564 | 1.48E-05 | 0.000115 | + | 0.432449786 | thioredoxin domain containing 12(TXNDC12) | UP_KW_DOMAIN: Redox-active center |
| ENSG00000125827 | TMX4 | protein_coding | -0.09516 | 4.745869 | 1.36E-06 | 1.67E-05 | + | 0.51050288 | thioredoxin related transmembrane protein 4(TMX4) | UP_KW_DOMAIN: Redox-active center |
| ENSG00000136810 | TXN | protein_coding | -0.11373 | 6.08377 | 4.54E-06 | 4.42E-05 | + | 0.602764553 | thioredoxin(TXN) | UP_KW_DOMAIN: Redox-active center |
| ENSG00000139921 | TMX1 | protein_coding | -0.11821 | 3.705677 | 5.91E-10 | 3.86E-08 | + | 0.500311417 | thioredoxin related transmembrane protein 1(TMX1) | UP_KW_DOMAIN: Redox-active center |
| ENSG00000167004 | PDIA3 | protein_coding | -0.13295 | 8.332991 | 6.42E-07 | 9.01E-06 | + | 0.48431909 | protein disulfide isomerase family A member 3(PDIA3) | UP_KW_DOMAIN: Redox-active center |
| ENSG00000197930 | ERO1L | protein_coding | -0.09029 | 4.89313 | 2.05E-05 | 0.000151 | + | 0.55642251 | endoplasmic reticulum oxidoreductase 1 alpha(ERO1A) | UP_KW_DOMAIN: Redox-active center |
| ENSG00000198431 | TXNRD1 | protein_coding | -0.06789 | 5.876409 | 0.000301 | 0.001335 | + | 0.570508663 | thioredoxin reductase 1(TXNRD1) | UP_KW_DOMAIN: Redox-active center |
| ENSG00000198843 | SELT | protein_coding | -0.07531 | 3.37023 | 0.00132 | 0.004464 | + | 0.396188449 | selenoprotein T(SELENOT) | UP_KW_DOMAIN: Redox-active center |
| ENSG00000216490 | IFI30 | protein_coding | -0.08884 | 4.488374 | 0.002116 | 0.006588 | + | 0.40903337 | IFI30 lysosomal thiol reductase(IFI30) | UP_KW_DOMAIN: Redox-active center |
| ENSG00000099949 | LZTR1 | protein_coding | 0.068952 | 4.532476 | 2.89E-05 | 0.0002 | - | -0.460132731 | leucine zipper like post translational regulator 1(LZTR1) | Smart: Kelch |
| ENSG00000117153 | KLHL12 | protein_coding | 0.097473 | 4.427338 | 5.11E-08 | 1.22E-06 | - | -0.481429777 | kelch like family member 12(KLHL12) | Smart: Kelch |
| ENSG00000120696 | KBTBD7 | protein_coding | 0.072658 | 3.836226 | 4.26E-05 | 0.000274 | - | -0.404423283 | kelch repeat and BTB domain containing 7(KBTBD7) | Smart: Kelch |
| ENSG00000128607 | KLHDC10 | protein_coding | 0.105188 | 5.770583 | 1.55E-07 | 2.94E-06 | - | -0.540219952 | kelch domain containing 10(KLHDC10) | Smart: Kelch |
| ENSG00000146021 | KLHL3 | protein_coding | 0.159994 | 2.930541 | 2.2E-07 | 3.87E-06 | - | -0.492214002 | kelch like family member 3(KLHL3) | Smart: Kelch |
| ENSG00000179023 | KLHDC7A | protein_coding | 0.275304 | 0.623788 | 1.91E-08 | 5.63E-07 | - | -0.495144522 | kelch domain containing 7A(KLHDC7A) | Smart: Kelch |
| ENSG00000187961 | KLHL17 | protein_coding | 0.186831 | 0.606556 | 8.83E-05 | 0.000491 | - | -0.455869462 | kelch like family member 17(KLHL17) | Smart: Kelch |
| ENSG00000100292 | HMOX1 | protein_coding | -0.15605 | 5.518844 | 2.22E-06 | 2.46E-05 | + | 0.54498319 | HMOX1 | Oxidative Stress Induced Gene Expression Via Nrf2 |
| ENSG00000103415 | HMOX2 | protein_coding | -0.19526 | 3.193561 | 4.71E-11 | 5.81E-09 | + | 0.491554623 | HMOX2 | Oxidative Stress Induced Gene Expression Via Nrf2 |
| ENSG00000116044 | NFE2L2 | protein_coding | -0.09094 | 6.286134 | 8.82E-11 | 9.02E-09 | + | 1 | NFE2L2 | Oxidative Stress Induced Gene Expression Via Nrf2 |
| ENSG00000127948 | POR | protein_coding | -0.14622 | 5.011253 | 2.64E-06 | 2.84E-05 | + | 0.381189712 | POR | Oxidative Stress Induced Gene Expression Via Nrf2 |
| ENSG00000154229 | PRKCA | protein_coding | 0.08646 | 5.498978 | 1.58E-06 | 1.88E-05 | - | -0.529209289 | PRKCA | Oxidative Stress Induced Gene Expression Via Nrf2 |
| ENSG00000170345 | FOS | protein_coding | -0.93273 | 2.703951 | 2.08E-17 | 5.99E-14 | + | 0.636507656 | FOS | Oxidative Stress Induced Gene Expression Via Nrf2 |
| ENSG00000177606 | JUN | protein_coding | -0.54709 | 3.15735 | 3.43E-12 | 7.49E-10 | + | 0.614988547 | JUN | Oxidative Stress Induced Gene Expression Via Nrf2 |
| ENSG00000185022 | MAFF | protein_coding | -0.28922 | -1.13315 | 4.76E-06 | 4.6E-05 | + | 0.469294214 | MAFF | Oxidative Stress Induced Gene Expression Via Nrf2 |
| ENSG00000004779 | NDUFAB1 | protein_coding | -0.06613 | 4.019654 | 0.002445 | 0.007429 | + | 0.424879803 | NADH:ubiquinone oxidoreductase subunit AB1(NDUFAB1) | KEGG: Oxidative phosphorylation |
| ENSG00000065518 | NDUFB4 | protein_coding | -0.11521 | 4.616882 | 1.09E-07 | 2.21E-06 | + | 0.416072625 | NADH:ubiquinone oxidoreductase subunit B4(NDUFB4) | KEGG: Oxidative phosphorylation |
| ENSG00000110719 | TCIRG1 | protein_coding | 0.066608 | 5.346511 | 9.15E-05 | 0.000505 | - | -0.490212177 | T cell immune regulator 1, ATPase H+ transporting V0 subunit a3(TCIRG1) | KEGG: Oxidative phosphorylation |
| ENSG00000113732 | ATP6V0E1 | protein_coding | -0.14996 | 4.323916 | 2.17E-11 | 3.04E-09 | + | 0.53995053 | ATPase H+ transporting V0 subunit e1(ATP6V0E1) | KEGG: Oxidative phosphorylation |
| ENSG00000116459 | ATP5F1 | protein_coding | -0.08151 | 4.387317 | 7.91E-05 | 0.000449 | + | 0.426398007 | ATP synthase peripheral stalk-membrane subunit b(ATP5PB) | KEGG: Oxidative phosphorylation |
| ENSG00000124172 | ATP5E | protein_coding | -0.11623 | 6.293449 | 4.82E-07 | 7.16E-06 | + | 0.463529585 | ATP synthase F1 subunit epsilon(ATP5F1E) | KEGG: Oxidative phosphorylation |
| ENSG00000125356 | NDUFA1 | protein_coding | -0.1116 | 6.244789 | 5.86E-06 | 5.47E-05 | + | 0.435009824 | NADH:ubiquinone oxidoreductase subunit A1(NDUFA1) | KEGG: Oxidative phosphorylation |
| ENSG00000127184 | COX7C | protein_coding | -0.12535 | 5.584793 | 1.13E-09 | 6.23E-08 | + | 0.581256474 | cytochrome c oxidase subunit 7C(COX7C) | KEGG: Oxidative phosphorylation |
| ENSG00000128609 | NDUFA5 | protein_coding | -0.08207 | 4.327593 | 6.55E-06 | 5.98E-05 | + | 0.504089407 | NADH:ubiquinone oxidoreductase subunit A5(NDUFA5) | KEGG: Oxidative phosphorylation |
| ENSG00000131100 | ATP6V1E1 | protein_coding | -0.13318 | 3.948518 | 4.22E-07 | 6.44E-06 | + | 0.460721079 | ATPase H+ transporting V1 subunit E1(ATP6V1E1) | KEGG: Oxidative phosphorylation |
| ENSG00000131143 | COX4I1 | protein_coding | -0.09535 | 5.44147 | 7.1E-07 | 9.82E-06 | + | 0.463433684 | cytochrome c oxidase subunit 4I1(COX4I1) | KEGG: Oxidative phosphorylation |
| ENSG00000131174 | COX7B | protein_coding | -0.11171 | 3.918265 | 0.001645 | 0.005341 | + | 0.401360612 | cytochrome c oxidase subunit 7B(COX7B) | KEGG: Oxidative phosphorylation |
| ENSG00000136521 | NDUFB5 | protein_coding | -0.07014 | 5.248537 | 5.39E-07 | 7.81E-06 | + | 0.439337025 | NADH:ubiquinone oxidoreductase subunit B5(NDUFB5) | KEGG: Oxidative phosphorylation |
| ENSG00000138495 | COX17 | protein_coding | -0.1267 | 3.102811 | 2.1E-08 | 6.09E-07 | + | 0.486489395 | cytochrome c oxidase copper chaperone COX17(COX17) | KEGG: Oxidative phosphorylation |
| ENSG00000154723 | ATP5J | protein_coding | -0.11668 | 4.79327 | 4.64E-08 | 1.12E-06 | + | 0.47874522 | ATP synthase peripheral stalk subunit F6(ATP5PF) | KEGG: Oxidative phosphorylation |
| ENSG00000155097 | ATP6V1C1 | protein_coding | -0.11224 | 4.802629 | 4.19E-07 | 6.4E-06 | + | 0.596045472 | ATPase H+ transporting V1 subunit C1(ATP6V1C1) | KEGG: Oxidative phosphorylation |
| ENSG00000156467 | UQCRB | protein_coding | -0.05709 | 7.601628 | 0.002745 | 0.00818 | + | 0.415266289 | ubiquinol-cytochrome c reductase binding protein(UQCRB) | KEGG: Oxidative phosphorylation |
| ENSG00000164405 | UQCRQ | protein_coding | -0.08199 | 5.779706 | 0.000101 | 0.000549 | + | 0.445150625 | ubiquinol-cytochrome c reductase complex III subunit VII(UQCRQ) | KEGG: Oxidative phosphorylation |
| ENSG00000165264 | NDUFB6 | protein_coding | -0.1616 | 2.452408 | 4.35E-05 | 0.000278 | + | 0.453488461 | NADH:ubiquinone oxidoreductase subunit B6(NDUFB6) | KEGG: Oxidative phosphorylation |
| ENSG00000165629 | ATP5C1 | protein_coding | -0.09851 | 6.297069 | 3.34E-08 | 8.9E-07 | + | 0.482972105 | ATP synthase F1 subunit gamma(ATP5F1C) | KEGG: Oxidative phosphorylation |
| ENSG00000166260 | COX11 | protein_coding | 0.047281 | 5.166712 | 0.001643 | 0.005335 | - | -0.463019309 | cytochrome c oxidase copper chaperone COX11(COX11) | KEGG: Oxidative phosphorylation |
| ENSG00000167283 | ATP5L | protein_coding | -0.13188 | 4.241409 | 2.66E-06 | 2.85E-05 | + | 0.43831502 | ATP synthase membrane subunit g(ATP5MG) | KEGG: Oxidative phosphorylation |
| ENSG00000167863 | ATP5H | protein_coding | -0.11637 | 5.20976 | 3.05E-06 | 3.17E-05 | + | 0.405089406 | ATP synthase peripheral stalk subunit d(ATP5PD) | KEGG: Oxidative phosphorylation |
| ENSG00000168653 | NDUFS5 | protein_coding | -0.13181 | 4.571444 | 4.37E-05 | 0.000279 | + | 0.390541373 | NADH:ubiquinone oxidoreductase subunit S5(NDUFS5) | KEGG: Oxidative phosphorylation |
| ENSG00000172115 | CYCS | protein_coding | -0.13059 | 4.585726 | 0.001375 | 0.0046 | + | 0.424173919 | cytochrome c, somatic(CYCS) | KEGG: Oxidative phosphorylation |
| ENSG00000178127 | NDUFV2 | protein_coding | -0.06421 | 6.999764 | 0.000319 | 0.0014 | + | 0.420958132 | NADH:ubiquinone oxidoreductase core subunit V2(NDUFV2) | KEGG: Oxidative phosphorylation |
| ENSG00000180817 | PPA1 | protein_coding | -0.13111 | 5.525066 | 4.74E-07 | 7.06E-06 | + | 0.611874949 | inorganic pyrophosphatase 1(PPA1) | KEGG: Oxidative phosphorylation |
| ENSG00000183648 | NDUFB1 | protein_coding | -0.07402 | 6.086949 | 0.001292 | 0.004386 | + | 0.401039894 | NADH:ubiquinone oxidoreductase subunit B1(NDUFB1) | KEGG: Oxidative phosphorylation |
| ENSG00000184076 | UQCR10 | protein_coding | -0.09638 | 4.416914 | 2.15E-05 | 0.000156 | + | 0.448526942 | ubiquinol-cytochrome c reductase, complex III subunit X(UQCR10) | KEGG: Oxidative phosphorylation |
| ENSG00000189043 | NDUFA4 | protein_coding | -0.11111 | 6.464156 | 7.12E-06 | 6.39E-05 | + | 0.435477006 | NDUFA4 mitochondrial complex associated(NDUFA4) | KEGG: Oxidative phosphorylation |
| ENSG00000241837 | ATP5O | protein_coding | -0.10222 | 5.668911 | 1.32E-06 | 1.63E-05 | + | 0.455971124 | ATP synthase peripheral stalk subunit OSCP(ATP5PO) | KEGG: Oxidative phosphorylation |
| ENSG00000004779 | NDUFAB1 | protein_coding | -0.06613 | 4.019654 | 0.002445 | 0.007429 | + | 0.424879803 | NADH:ubiquinone oxidoreductase subunit AB1(NDUFAB1) | KEGG: Non-alcoholic fatty liver disease |
| ENSG00000009950 | MLXIPL | protein_coding | 0.143404 | 7.33379 | 6.08E-08 | 1.4E-06 | - | -0.5687726 | MLX interacting protein like(MLXIPL) | KEGG: Non-alcoholic fatty liver disease |
| ENSG00000025434 | NR1H3 | protein_coding | 0.083052 | 4.704539 | 4.38E-05 | 0.000279 | - | -0.563777424 | nuclear receptor subfamily 1 group H member 3(NR1H3) | KEGG: Non-alcoholic fatty liver disease |
| ENSG00000065518 | NDUFB4 | protein_coding | -0.11521 | 4.616882 | 1.09E-07 | 2.21E-06 | + | 0.416072625 | NADH:ubiquinone oxidoreductase subunit B4(NDUFB4) | KEGG: Non-alcoholic fatty liver disease |
| ENSG00000067182 | TNFRSF1A | protein_coding | -0.1191 | 6.355421 | 8.73E-09 | 3.12E-07 | + | 0.554168229 | TNF receptor superfamily member 1A(TNFRSF1A) | KEGG: Non-alcoholic fatty liver disease |
| ENSG00000070831 | CDC42 | protein_coding | -0.13949 | 5.054292 | 1.03E-08 | 3.55E-07 | + | 0.619306405 | cell division cycle 42(CDC42) | KEGG: Non-alcoholic fatty liver disease |
| ENSG00000104365 | IKBKB | protein_coding | 0.064881 | 5.41767 | 1.44E-06 | 1.75E-05 | - | -0.458560932 | inhibitor of nuclear factor kappa B kinase subunit beta(IKBKB) | KEGG: Non-alcoholic fatty liver disease |
| ENSG00000105221 | AKT2 | protein_coding | 0.056381 | 6.820179 | 0.000455 | 0.001868 | - | -0.458560162 | AKT serine/threonine kinase 2(AKT2) | KEGG: Non-alcoholic fatty liver disease |
| ENSG00000105329 | TGFB1 | protein_coding | -0.06165 | 3.966073 | 0.002129 | 0.006624 | + | 0.406790511 | transforming growth factor beta 1(TGFB1) | KEGG: Non-alcoholic fatty liver disease |
| ENSG00000109320 | NFKB1 | protein_coding | -0.0912 | 5.468011 | 3.82E-08 | 9.75E-07 | + | 0.561511709 | nuclear factor kappa B subunit 1(NFKB1) | KEGG: Non-alcoholic fatty liver disease |
| ENSG00000115592 | PRKAG3 | protein_coding | 0.208438 | 1.302705 | 0.003342 | 0.009609 | - | -0.52486897 | protein kinase AMP-activated non-catalytic subunit gamma 3(PRKAG3) | KEGG: Non-alcoholic fatty liver disease |
| ENSG00000116678 | LEPR | protein_coding | -0.15216 | 8.193728 | 1.03E-06 | 1.33E-05 | + | 0.480108923 | leptin receptor(LEPR) | KEGG: Non-alcoholic fatty liver disease |
| ENSG00000125356 | NDUFA1 | protein_coding | -0.1116 | 6.244789 | 5.86E-06 | 5.47E-05 | + | 0.435009824 | NADH:ubiquinone oxidoreductase subunit A1(NDUFA1) | KEGG: Non-alcoholic fatty liver disease |
| ENSG00000127184 | COX7C | protein_coding | -0.12535 | 5.584793 | 1.13E-09 | 6.23E-08 | + | 0.581256474 | cytochrome c oxidase subunit 7C(COX7C) | KEGG: Non-alcoholic fatty liver disease |
| ENSG00000128609 | NDUFA5 | protein_coding | -0.08207 | 4.327593 | 6.55E-06 | 5.98E-05 | + | 0.504089407 | NADH:ubiquinone oxidoreductase subunit A5(NDUFA5) | KEGG: Non-alcoholic fatty liver disease |
| ENSG00000131143 | COX4I1 | protein_coding | -0.09535 | 5.44147 | 7.1E-07 | 9.82E-06 | + | 0.463433684 | cytochrome c oxidase subunit 4I1(COX4I1) | KEGG: Non-alcoholic fatty liver disease |
| ENSG00000131174 | COX7B | protein_coding | -0.11171 | 3.918265 | 0.001645 | 0.005341 | + | 0.401360612 | cytochrome c oxidase subunit 7B(COX7B) | KEGG: Non-alcoholic fatty liver disease |
| ENSG00000134001 | EIF2S1 | protein_coding | -0.17655 | 4.244157 | 1.86E-10 | 1.61E-08 | + | 0.555795528 | eukaryotic translation initiation factor 2 subunit alpha(EIF2S1) | KEGG: Non-alcoholic fatty liver disease |
| ENSG00000136238 | RAC1 | protein_coding | -0.20657 | 3.920685 | 2.64E-15 | 3.04E-12 | + | 0.634848587 | Rac family small GTPase 1(RAC1) | KEGG: Non-alcoholic fatty liver disease |
| ENSG00000136244 | IL6 | protein_coding | -0.43811 | -3.48338 | 1.51E-07 | 2.87E-06 | + | 0.560821802 | interleukin 6(IL6) | KEGG: Non-alcoholic fatty liver disease |
| ENSG00000136521 | NDUFB5 | protein_coding | -0.07014 | 5.248537 | 5.39E-07 | 7.81E-06 | + | 0.439337025 | NADH:ubiquinone oxidoreductase subunit B5(NDUFB5) | KEGG: Non-alcoholic fatty liver disease |
| ENSG00000156467 | UQCRB | protein_coding | -0.05709 | 7.601628 | 0.002745 | 0.00818 | + | 0.415266289 | ubiquinol-cytochrome c reductase binding protein(UQCRB) | KEGG: Non-alcoholic fatty liver disease |
| ENSG00000159346 | ADIPOR1 | protein_coding | -0.07871 | 5.199359 | 2.16E-06 | 2.41E-05 | + | 0.391835733 | adiponectin receptor 1(ADIPOR1) | KEGG: Non-alcoholic fatty liver disease |
| ENSG00000164405 | UQCRQ | protein_coding | -0.08199 | 5.779706 | 0.000101 | 0.000549 | + | 0.445150625 | ubiquinol-cytochrome c reductase complex III subunit VII(UQCRQ) | KEGG: Non-alcoholic fatty liver disease |
| ENSG00000165264 | NDUFB6 | protein_coding | -0.1616 | 2.452408 | 4.35E-05 | 0.000278 | + | 0.453488461 | NADH:ubiquinone oxidoreductase subunit B6(NDUFB6) | KEGG: Non-alcoholic fatty liver disease |
| ENSG00000168653 | NDUFS5 | protein_coding | -0.13181 | 4.571444 | 4.37E-05 | 0.000279 | + | 0.390541373 | NADH:ubiquinone oxidoreductase subunit S5(NDUFS5) | KEGG: Non-alcoholic fatty liver disease |
| ENSG00000169429 | IL8 | protein_coding | -0.51591 | -0.28481 | 4.54E-07 | 6.82E-06 | + | 0.558951209 | C-X-C motif chemokine ligand 8(CXCL8) | KEGG: Non-alcoholic fatty liver disease |
| ENSG00000170345 | FOS | protein_coding | -0.93273 | 2.703951 | 2.08E-17 | 5.99E-14 | + | 0.636507656 | Fos proto-oncogene, AP-1 transcription factor subunit(FOS) | KEGG: Non-alcoholic fatty liver disease |
| ENSG00000172115 | CYCS | protein_coding | -0.13059 | 4.585726 | 0.001375 | 0.0046 | + | 0.424173919 | cytochrome c, somatic(CYCS) | KEGG: Non-alcoholic fatty liver disease |
| ENSG00000173327 | MAP3K11 | protein_coding | 0.061003 | 3.658902 | 0.001334 | 0.004501 | - | -0.417510659 | mitogen-activated protein kinase kinase kinase 11(MAP3K11) | KEGG: Non-alcoholic fatty liver disease |
| ENSG00000177606 | JUN | protein_coding | -0.54709 | 3.15735 | 3.43E-12 | 7.49E-10 | + | 0.614988547 | Jun proto-oncogene, AP-1 transcription factor subunit(JUN) | KEGG: Non-alcoholic fatty liver disease |
| ENSG00000178127 | NDUFV2 | protein_coding | -0.06421 | 6.999764 | 0.000319 | 0.0014 | + | 0.420958132 | NADH:ubiquinone oxidoreductase core subunit V2(NDUFV2) | KEGG: Non-alcoholic fatty liver disease |
| ENSG00000178607 | ERN1 | protein_coding | -0.11447 | 5.024652 | 1.61E-07 | 3.02E-06 | + | 0.414312125 | endoplasmic reticulum to nucleus signaling 1(ERN1) | KEGG: Non-alcoholic fatty liver disease |
| ENSG00000183648 | NDUFB1 | protein_coding | -0.07402 | 6.086949 | 0.001292 | 0.004386 | + | 0.401039894 | NADH:ubiquinone oxidoreductase subunit B1(NDUFB1) | KEGG: Non-alcoholic fatty liver disease |
| ENSG00000184076 | UQCR10 | protein_coding | -0.09638 | 4.416914 | 2.15E-05 | 0.000156 | + | 0.448526942 | ubiquinol-cytochrome c reductase, complex III subunit X(UQCR10) | KEGG: Non-alcoholic fatty liver disease |
| ENSG00000184557 | SOCS3 | protein_coding | -0.6636 | -1.10138 | 6.92E-13 | 2.36E-10 | + | 0.595612194 | suppressor of cytokine signaling 3(SOCS3) | KEGG: Non-alcoholic fatty liver disease |
| ENSG00000186350 | RXRA | protein_coding | 0.087178 | 6.100088 | 0.001204 | 0.004138 | - | -0.503042732 | retinoid X receptor alpha(RXRA) | KEGG: Non-alcoholic fatty liver disease |
| ENSG00000186951 | PPARA | protein_coding | 0.078771 | 7.044694 | 0.001469 | 0.004858 | - | -0.473489077 | peroxisome proliferator activated receptor alpha(PPARA) | KEGG: Non-alcoholic fatty liver disease |
| ENSG00000188130 | MAPK12 | protein_coding | 0.064528 | 3.396187 | 0.000852 | 0.003111 | - | -0.391143996 | mitogen-activated protein kinase 12(MAPK12) | KEGG: Non-alcoholic fatty liver disease |
| ENSG00000189043 | NDUFA4 | protein_coding | -0.11111 | 6.464156 | 7.12E-06 | 6.39E-05 | + | 0.435477006 | NDUFA4 mitochondrial complex associated(NDUFA4) | KEGG: Non-alcoholic fatty liver disease |
| ENSG00000245848 | CEBPA | protein_coding | 0.19325 | 4.731397 | 1.98E-05 | 0.000146 | - | -0.411893612 | CCAAT enhancer binding protein alpha(CEBPA) | KEGG: Non-alcoholic fatty liver disease |
| ENSG00000254647 | INS | protein_coding | 0.339956 | 0.169299 | 2.81E-07 | 4.69E-06 | - | -0.654548642 | insulin(INS) | KEGG: Non-alcoholic fatty liver disease |
| ENSG00000004779 | NDUFAB1 | protein_coding | -0.06613 | 4.019654 | 0.002445 | 0.007429 | + | 0.424879803 | NADH:ubiquinone oxidoreductase subunit AB1(NDUFAB1) | KEGG: Chemical carcinogenesis - reactive oxygen species |
| ENSG00000019991 | HGF | protein_coding | -0.0678 | 6.191604 | 0.001111 | 0.003881 | + | 0.448981405 | hepatocyte growth factor(HGF) | KEGG: Chemical carcinogenesis - reactive oxygen species |
| ENSG00000065518 | NDUFB4 | protein_coding | -0.11521 | 4.616882 | 1.09E-07 | 2.21E-06 | + | 0.416072625 | NADH:ubiquinone oxidoreductase subunit B4(NDUFB4) | KEGG: Chemical carcinogenesis - reactive oxygen species |
| ENSG00000065559 | MAP2K4 | protein_coding | -0.09755 | 4.040945 | 2.55E-09 | 1.19E-07 | + | 0.608923844 | mitogen-activated protein kinase kinase 4(MAP2K4) | KEGG: Chemical carcinogenesis - reactive oxygen species |
| ENSG00000075651 | PLD1 | protein_coding | 0.087182 | 6.602159 | 8.25E-07 | 1.1E-05 | - | -0.417744097 | phospholipase D1(PLD1) | KEGG: Chemical carcinogenesis - reactive oxygen species |
| ENSG00000100292 | HMOX1 | protein_coding | -0.15605 | 5.518844 | 2.22E-06 | 2.46E-05 | + | 0.54498319 | heme oxygenase 1(HMOX1) | KEGG: Chemical carcinogenesis - reactive oxygen species |
| ENSG00000100644 | HIF1A | protein_coding | -0.07926 | 6.679924 | 0.000369 | 0.001577 | + | 0.613037092 | hypoxia inducible factor 1 subunit alpha(HIF1A) | KEGG: Chemical carcinogenesis - reactive oxygen species |
| ENSG00000100906 | NFKBIA | protein_coding | -0.13494 | 7.249689 | 5.5E-08 | 1.29E-06 | + | 0.579136593 | NFKB inhibitor alpha(NFKBIA) | KEGG: Chemical carcinogenesis - reactive oxygen species |
| ENSG00000104365 | IKBKB | protein_coding | 0.064881 | 5.41767 | 1.44E-06 | 1.75E-05 | - | -0.458560932 | inhibitor of nuclear factor kappa B kinase subunit beta(IKBKB) | KEGG: Chemical carcinogenesis - reactive oxygen species |
| ENSG00000105221 | AKT2 | protein_coding | 0.056381 | 6.820179 | 0.000455 | 0.001868 | - | -0.458560162 | AKT serine/threonine kinase 2(AKT2) | KEGG: Chemical carcinogenesis - reactive oxygen species |
| ENSG00000106546 | AHR | protein_coding | -0.04895 | 7.098888 | 0.002654 | 0.007955 | + | 0.46442872 | aryl hydrocarbon receptor(AHR) | KEGG: Chemical carcinogenesis - reactive oxygen species |
| ENSG00000109320 | NFKB1 | protein_coding | -0.0912 | 5.468011 | 3.82E-08 | 9.75E-07 | + | 0.561511709 | nuclear factor kappa B subunit 1(NFKB1) | KEGG: Chemical carcinogenesis - reactive oxygen species |
| ENSG00000116044 | NFE2L2 | protein_coding | -0.09094 | 6.286134 | 8.82E-11 | 9.02E-09 | + | 1 | NFE2 like bZIP transcription factor 2(NFE2L2) | KEGG: Chemical carcinogenesis - reactive oxygen species |
| ENSG00000116459 | ATP5F1 | protein_coding | -0.08151 | 4.387317 | 7.91E-05 | 0.000449 | + | 0.426398007 | ATP synthase peripheral stalk-membrane subunit b(ATP5PB) | KEGG: Chemical carcinogenesis - reactive oxygen species |
| ENSG00000118689 | FOXO3 | protein_coding | -0.17476 | 3.141256 | 5.86E-11 | 6.77E-09 | + | 0.452477762 | forkhead box O3(FOXO3) | KEGG: Chemical carcinogenesis - reactive oxygen species |
| ENSG00000124172 | ATP5E | protein_coding | -0.11623 | 6.293449 | 4.82E-07 | 7.16E-06 | + | 0.463529585 | ATP synthase F1 subunit epsilon(ATP5F1E) | KEGG: Chemical carcinogenesis - reactive oxygen species |
| ENSG00000125356 | NDUFA1 | protein_coding | -0.1116 | 6.244789 | 5.86E-06 | 5.47E-05 | + | 0.435009824 | NADH:ubiquinone oxidoreductase subunit A1(NDUFA1) | KEGG: Chemical carcinogenesis - reactive oxygen species |
| ENSG00000127184 | COX7C | protein_coding | -0.12535 | 5.584793 | 1.13E-09 | 6.23E-08 | + | 0.581256474 | cytochrome c oxidase subunit 7C(COX7C) | KEGG: Chemical carcinogenesis - reactive oxygen species |
| ENSG00000128609 | NDUFA5 | protein_coding | -0.08207 | 4.327593 | 6.55E-06 | 5.98E-05 | + | 0.504089407 | NADH:ubiquinone oxidoreductase subunit A5(NDUFA5) | KEGG: Chemical carcinogenesis - reactive oxygen species |
| ENSG00000129219 | PLD2 | protein_coding | 0.073968 | 3.716198 | 7.5E-05 | 0.000432 | - | -0.435140742 | phospholipase D2(PLD2) | KEGG: Chemical carcinogenesis - reactive oxygen species |
| ENSG00000131143 | COX4I1 | protein_coding | -0.09535 | 5.44147 | 7.1E-07 | 9.82E-06 | + | 0.463433684 | cytochrome c oxidase subunit 4I1(COX4I1) | KEGG: Chemical carcinogenesis - reactive oxygen species |
| ENSG00000131174 | COX7B | protein_coding | -0.11171 | 3.918265 | 0.001645 | 0.005341 | + | 0.401360612 | cytochrome c oxidase subunit 7B(COX7B) | KEGG: Chemical carcinogenesis - reactive oxygen species |
| ENSG00000134202 | GSTM3 | protein_coding | -0.23585 | 1.829259 | 1.65E-06 | 1.94E-05 | + | 0.400868603 | glutathione S-transferase mu 3(GSTM3) | KEGG: Chemical carcinogenesis - reactive oxygen species |
| ENSG00000136238 | RAC1 | protein_coding | -0.20657 | 3.920685 | 2.64E-15 | 3.04E-12 | + | 0.634848587 | Rac family small GTPase 1(RAC1) | KEGG: Chemical carcinogenesis - reactive oxygen species |
| ENSG00000136521 | NDUFB5 | protein_coding | -0.07014 | 5.248537 | 5.39E-07 | 7.81E-06 | + | 0.439337025 | NADH:ubiquinone oxidoreductase subunit B5(NDUFB5) | KEGG: Chemical carcinogenesis - reactive oxygen species |
| ENSG00000140992 | PDPK1 | protein_coding | 0.089537 | 3.768991 | 0.001545 | 0.005064 | - | -0.39071729 | 3-phosphoinositide dependent protein kinase 1(PDPK1) | KEGG: Chemical carcinogenesis - reactive oxygen species |
| ENSG00000145217 | SLC26A1 | protein_coding | 0.127091 | 2.641935 | 5.87E-05 | 0.000354 | - | -0.479931867 | solute carrier family 26 member 1(SLC26A1) | KEGG: Chemical carcinogenesis - reactive oxygen species |
| ENSG00000148834 | GSTO1 | protein_coding | -0.11873 | 6.152212 | 1.77E-06 | 2.06E-05 | + | 0.408565819 | glutathione S-transferase omega 1(GSTO1) | KEGG: Chemical carcinogenesis - reactive oxygen species |
| ENSG00000151729 | SLC25A4 | protein_coding | -0.15018 | 4.217883 | 2.68E-09 | 1.23E-07 | + | 0.490948013 | solute carrier family 25 member 4(SLC25A4) | KEGG: Chemical carcinogenesis - reactive oxygen species |
| ENSG00000154723 | ATP5J | protein_coding | -0.11668 | 4.79327 | 4.64E-08 | 1.12E-06 | + | 0.47874522 | ATP synthase peripheral stalk subunit F6(ATP5PF) | KEGG: Chemical carcinogenesis - reactive oxygen species |
| ENSG00000156467 | UQCRB | protein_coding | -0.05709 | 7.601628 | 0.002745 | 0.00818 | + | 0.415266289 | ubiquinol-cytochrome c reductase binding protein(UQCRB) | KEGG: Chemical carcinogenesis - reactive oxygen species |
| ENSG00000164405 | UQCRQ | protein_coding | -0.08199 | 5.779706 | 0.000101 | 0.000549 | + | 0.445150625 | ubiquinol-cytochrome c reductase complex III subunit VII(UQCRQ) | KEGG: Chemical carcinogenesis - reactive oxygen species |
| ENSG00000165264 | NDUFB6 | protein_coding | -0.1616 | 2.452408 | 4.35E-05 | 0.000278 | + | 0.453488461 | NADH:ubiquinone oxidoreductase subunit B6(NDUFB6) | KEGG: Chemical carcinogenesis - reactive oxygen species |
| ENSG00000165629 | ATP5C1 | protein_coding | -0.09851 | 6.297069 | 3.34E-08 | 8.9E-07 | + | 0.482972105 | ATP synthase F1 subunit gamma(ATP5F1C) | KEGG: Chemical carcinogenesis - reactive oxygen species |
| ENSG00000165637 | VDAC2 | protein_coding | -0.12069 | 3.226872 | 2.87E-07 | 4.75E-06 | + | 0.555762403 | voltage dependent anion channel 2(VDAC2) | KEGG: Chemical carcinogenesis - reactive oxygen species |
| ENSG00000167863 | ATP5H | protein_coding | -0.11637 | 5.20976 | 3.05E-06 | 3.17E-05 | + | 0.405089406 | ATP synthase peripheral stalk subunit d(ATP5PD) | KEGG: Chemical carcinogenesis - reactive oxygen species |
| ENSG00000168653 | NDUFS5 | protein_coding | -0.13181 | 4.571444 | 4.37E-05 | 0.000279 | + | 0.390541373 | NADH:ubiquinone oxidoreductase subunit S5(NDUFS5) | KEGG: Chemical carcinogenesis - reactive oxygen species |
| ENSG00000170345 | FOS | protein_coding | -0.93273 | 2.703951 | 2.08E-17 | 5.99E-14 | + | 0.636507656 | Fos proto-oncogene, AP-1 transcription factor subunit(FOS) | KEGG: Chemical carcinogenesis - reactive oxygen species |
| ENSG00000177606 | JUN | protein_coding | -0.54709 | 3.15735 | 3.43E-12 | 7.49E-10 | + | 0.614988547 | Jun proto-oncogene, AP-1 transcription factor subunit(JUN) | KEGG: Chemical carcinogenesis - reactive oxygen species |
| ENSG00000178127 | NDUFV2 | protein_coding | -0.06421 | 6.999764 | 0.000319 | 0.0014 | + | 0.420958132 | NADH:ubiquinone oxidoreductase core subunit V2(NDUFV2) | KEGG: Chemical carcinogenesis - reactive oxygen species |
| ENSG00000183648 | NDUFB1 | protein_coding | -0.07402 | 6.086949 | 0.001292 | 0.004386 | + | 0.401039894 | NADH:ubiquinone oxidoreductase subunit B1(NDUFB1) | KEGG: Chemical carcinogenesis - reactive oxygen species |
| ENSG00000184076 | UQCR10 | protein_coding | -0.09638 | 4.416914 | 2.15E-05 | 0.000156 | + | 0.448526942 | ubiquinol-cytochrome c reductase, complex III subunit X(UQCR10) | KEGG: Chemical carcinogenesis - reactive oxygen species |
| ENSG00000188130 | MAPK12 | protein_coding | 0.064528 | 3.396187 | 0.000852 | 0.003111 | - | -0.391143996 | mitogen-activated protein kinase 12(MAPK12) | KEGG: Chemical carcinogenesis - reactive oxygen species |
| ENSG00000189043 | NDUFA4 | protein_coding | -0.11111 | 6.464156 | 7.12E-06 | 6.39E-05 | + | 0.435477006 | NDUFA4 mitochondrial complex associated(NDUFA4) | KEGG: Chemical carcinogenesis - reactive oxygen species |
| ENSG00000196396 | PTPN1 | protein_coding | -0.12422 | 4.375407 | 4.62E-05 | 0.000291 | + | 0.416971531 | protein tyrosine phosphatase non-receptor type 1(PTPN1) | KEGG: Chemical carcinogenesis - reactive oxygen species |
| ENSG00000213585 | VDAC1 | protein_coding | -0.09715 | 5.556897 | 0.000222 | 0.001043 | + | 0.392361443 | voltage dependent anion channel 1(VDAC1) | KEGG: Chemical carcinogenesis - reactive oxygen species |
| ENSG00000225697 | SLC26A6 | protein_coding | 0.109749 | 2.607041 | 0.001429 | 0.004744 | - | -0.423011925 | solute carrier family 26 member 6(SLC26A6) | KEGG: Chemical carcinogenesis - reactive oxygen species |
| ENSG00000241837 | ATP5O | protein_coding | -0.10222 | 5.668911 | 1.32E-06 | 1.63E-05 | + | 0.455971124 | ATP synthase peripheral stalk subunit OSCP(ATP5PO) | KEGG: Chemical carcinogenesis - reactive oxygen species |
| ENSG00000124194 | GDAP1L1 | protein_coding | 0.17718 | 0.875313 | 0.000762 | 0.002851 | - | -0.431480693 | ganglioside induced differentiation associated protein 1 like 1(GDAP1L1) | GO_BP: glutathione metabolic |
| ENSG00000124767 | GLO1 | protein_coding | -0.12942 | 5.206046 | 1.09E-06 | 1.4E-05 | + | 0.467292592 | glyoxalase I(GLO1) | GO_BP: glutathione metabolic |
| ENSG00000134202 | GSTM3 | protein_coding | -0.23585 | 1.829259 | 1.65E-06 | 1.94E-05 | + | 0.400868603 | glutathione S-transferase mu 3(GSTM3) | GO_BP: glutathione metabolic |
| ENSG00000148834 | GSTO1 | protein_coding | -0.11873 | 6.152212 | 1.77E-06 | 2.06E-05 | + | 0.408565819 | glutathione S-transferase omega 1(GSTO1) | GO_BP: glutathione metabolic |
| ENSG00000213719 | CLIC1 | protein_coding | -0.15095 | 2.678493 | 8.51E-06 | 7.4E-05 | + | 0.45438084 | chloride intracellular channel 1(CLIC1) | GO_BP: glutathione metabolic |
| ENSG00000115520 | COQ10B | protein_coding | -0.11467 | 2.873806 | 7.04E-07 | 9.76E-06 | + | 0.560825838 | coenzyme Q10B(COQ10B) | GO_BP: cellular respiration |
| ENSG00000119508 | NR4A3 | protein_coding | -0.37551 | 1.480949 | 8.44E-10 | 5.08E-08 | + | 0.535412159 | nuclear receptor subfamily 4 group A member 3(NR4A3) | GO_BP: cellular respiration |
| ENSG00000127184 | COX7C | protein_coding | -0.12535 | 5.584793 | 1.13E-09 | 6.23E-08 | + | 0.581256474 | cytochrome c oxidase subunit 7C(COX7C) | GO_BP: cellular respiration |
| ENSG00000131143 | COX4I1 | protein_coding | -0.09535 | 5.44147 | 7.1E-07 | 9.82E-06 | + | 0.463433684 | cytochrome c oxidase subunit 4I1(COX4I1) | GO_BP: cellular respiration |
| ENSG00000131174 | COX7B | protein_coding | -0.11171 | 3.918265 | 0.001645 | 0.005341 | + | 0.401360612 | cytochrome c oxidase subunit 7B(COX7B) | GO_BP: cellular respiration |
| ENSG00000135469 | COQ10A | protein_coding | 0.098809 | 3.047168 | 0.001767 | 0.00566 | - | -0.437975726 | coenzyme Q10A(COQ10A) | GO_BP: cellular respiration |
| ENSG00000156467 | UQCRB | protein_coding | -0.05709 | 7.601628 | 0.002745 | 0.00818 | + | 0.415266289 | ubiquinol-cytochrome c reductase binding protein(UQCRB) | GO_BP: cellular respiration |
| ENSG00000164405 | UQCRQ | protein_coding | -0.08199 | 5.779706 | 0.000101 | 0.000549 | + | 0.445150625 | ubiquinol-cytochrome c reductase complex III subunit VII(UQCRQ) | GO_BP: cellular respiration |
| ENSG00000172115 | CYCS | protein_coding | -0.13059 | 4.585726 | 0.001375 | 0.0046 | + | 0.424173919 | cytochrome c, somatic(CYCS) | GO_BP: cellular respiration |
| ENSG00000184076 | UQCR10 | protein_coding | -0.09638 | 4.416914 | 2.15E-05 | 0.000156 | + | 0.448526942 | ubiquinol-cytochrome c reductase, complex III subunit X(UQCR10) | GO_BP: cellular respiration |
| ENSG00000189043 | NDUFA4 | protein_coding | -0.11111 | 6.464156 | 7.12E-06 | 6.39E-05 | + | 0.435477006 | NDUFA4 mitochondrial complex associated(NDUFA4) | GO_BP: cellular respiration |
| ENSG00000117592 | PRDX6 | protein_coding | -0.142 | 6.372893 | 2.42E-08 | 6.84E-07 | + | 0.434891289 | PRDX6 | Antioxidant: TXN-based system |
| ENSG00000198431 | TXNRD1 | protein_coding | -0.06789 | 5.876409 | 0.000301 | 0.001335 | + | 0.570508663 | TXNRD1 | Antioxidant: TXN-based system |
| ENSG00000271303 | SRXN1 | protein_coding | -0.10828 | 2.455068 | 0.001741 | 0.005589 | + | 0.42663047 | SRXN1 | Antioxidant: TXN-based system |
| ENSG00000023909 | GCLM | protein_coding | -0.18416 | 4.15374 | 8.07E-12 | 1.48E-09 | + | 0.653475454 | GCLM | Antioxidant: GSH-based system |
| ENSG00000100031 | GGT1 | protein_coding | 0.127084 | 4.944384 | 3.19E-06 | 3.3E-05 | - | -0.325495321 | GGT1 | Antioxidant: GSH-based system |
| ENSG00000173221 | GLRX | protein_coding | -0.09005 | 4.546137 | 0.0055 | 0.0145 | + | 0.387781814 | GLRX | Antioxidant: GSH-based system |

**Supplementary Table 4.** Lists of antibodies.

| Target | Vender and Catalog |
| --- | --- |
| Keap1 | Cell signaling technology, 8047  Cell signaling technology, 4678 |
| Nrf2 | Cell signaling technology, 12721 |
| p62 | Cell signaling technology, 8025 |
| LC3 | Abcam, ab51520 |
| GSS | Santacruz, sc-166882 |
| GCLM | Abcam, ab126704 |
| CAT | Abcam, ab16731 |
| SOD2 | Cusabio, CSB-PA021788LA01HU |
| HO-1 | Abcam, ab13243 |
| GSH | Abcam, ab19534 |
| NAPQI | Biorad, 160104 |
| CYP2E1 | Cusabio, PA0064 |
| MPO | R&D systems, AF3667 |
| CRP | Santacruz, sc-69770 |
| CDH1 | Abcam, ab6528  Abcam, ab16505 |
| Beta-actin | Thermofisher, MA1-140 |
| GAPDH | Cell signaling technology, 5174 |
| Histone H3 | Cell signaling technology, 4494s |
| His-tag | Thermofisher, MA1-21315 |
| SNO-Cys | Abcam, Ab50185 |
| NO-Tyr | Abcam, ab42789 |
| 4-HNE | Abcam, ab48506 |
| Prx-SO_3_ | Abfrontier, LF-PA0004 |
| NADPH | Biorbyt, orb11152 |
| GSSG | Virogen, 101-A100 |
| TNF-α | Santacruz, sc-52746 |
| IL-1β | Abcam, ab9722 |
| FASN | Thermo scientific, PA1-32355 |
| p-ACC | Cell signaling technology, 3661S |
| SREBP-1c | Thermo scientific, PA1-46142 |

**Supplementary Table 5.** Primer sequences used in the Real-time PCR.

| Target | Forward | Reverse |
| --- | --- | --- |
| *Keap1* | 5’-GAT CGG CTG CAC TGA ACG G-3’ | 5’-GGA CTC GCA GCG TAC GTT-3’ |
| *GSS* | 5’-GGC TCC AAA GCC CTG AAA CA-3’ | 5’-GAC ATT GAG AAC GTG TCG GTG-3’ |
| *GCLM* | 5’-GAG CTT CGG GAC TGT ATC CAA-3’ | 5’-CCA AAA CAT CTG GAA ACT CCC TG-3’ |
| *CAT* | 5’-ATG GTC ACC GGC ACA TGA AT-3’ | 5’-TTT TTG ATG CCC TGG TCG GT-3’ |
| *SOD2* | 5’-TCG CTT ACA GAT TGC TGC CT-3’ | 5’-GGT AGT AAG CGT GCT CCC AC-3’ |
| *HO1* | 5’-CAC TCT GGA GAT GAC ACC TGA G-3’ | 5’-GGT TTC CTC TGT CAG CAT GAC C-3’ |

**Supplementary Table 6.** Sequences information of 5` region of Keap1 from pcDNA3-HA2-Keap1 and pcDNA3-His-HA-Keap1.

|  |  | Sequence (5` to 3`) |
| --- | --- | --- |
| pcDNA3-HA2-Keap1 | DNA | GASACCCAAGCTATGTACCCATACGAKGTTCCTGASTAWGSGGGCTATCCCTATGACGTCCCGGACTATGCAAGCTTG |
|  | Feature | HA tag |
| pcDNA3-His-HA-Keap1 | DNA | GACACCCAAGCTGCCGCCATGGCACATCATCACCATCACCATGGATCTTATCCCTATGACGTCCCGGACTATGCA |
|  | Feature | Kozak sequence / His tag / GS linker / HA tag |

**Supplementary Table 7.** Sequences information from T495 to G514 of pcDNA3-HA2-Keap1 and pcDNA3-His-HA-Keap1.

|  |  | Sequence (5` to 3`) | GC ratio |
| --- | --- | --- | --- |
| pcDNA3-HA2-Keap1 | DNA | ACCATCCGAAGCGGGGCAGGCGTCTGCGTCCTGCACAACTGTATCTATGCTGCTGGGGGC | 63.33% |
|  | Protein | T I R S G A G V C V L H N C I Y A A G G |  |
| pcDNA3-His-HA-Keap1 | DNA | ACTATCAGATCTGGAGCAGGAGTCTGTGTCCTGCACAACTGTATCTACGCTGCTGGAGGT | 51.66% |
|  | Protein | T I R S G A G V C V L H N C I Y A A G G |  |

**Supplementary Table 8.** Primer list for Keap1 mutation.

| Primer list | Sequence (5` to 3`) |
| --- | --- |
| P1_HA_Keap1_1stPCR_F | TATCCCTATGACGTCCCGGACTATGCAGGATCTCAGCCAGATCCCAGGCCTAGC |
| P2_Keap1_1stPCR_R | GACTCCTGCTCCAGATCTGATAGTGTTCATTGCTGTGATCATTCGC |
| P3_His-HA Keap1_2ndPCR_F | CATCATCACCATCACCATGGATCTTATCCCTATGACGTCCCGGAC |
| P4_Keap1_WT_2ndPCR_R | CCAGCAGCGTAGATACAGTTGTGCAGGACACAGACTCCTGCTCCAGATCTG |
| P5_Keap1_C513A_2ndPCR_R | CCAGCAGCGTAGATACAGTTGTGCAGGACAGCGACTCCTGCTCCAGATCTG |
| P6_Keap1_C518A_2ndPCR_R | CCAGCAGCGTAGATAGCGTTGTGCAGGACACAGACTCCTGCTCCAGATCTG |
| P7_Keap1_C513-518A_2ndPCR_R | CCAGCAGCGTAGATAGCGTTGTGCAGGACAGCGACTCCTGCTCCAGATCTG |
| P8_pcDNA3_Kozak_His_R | GTGATGGTGATGATGTGCCATGGCGGCAGCTTGGGTSTCCCTATAGTGAG |
| P9_pcDNA3_Keap1_F | TATCTACGCTGCTGGAGGTTATGATGGTCAGGACCAGCTG |

To facilitate ligation-independent cloning, overlapping sequences are highlighted in magenta (part of the His tag) and cyan (part of the modified sequence). In yellow, the Kozak sequence is highlighted. Mutation sequences are shown in red color character.

**Supplementary Figure 1. Betaine promotes Nrf2 nuclear translocation in AML12 cells.**

Representative immunofluorescence images show Nrf2 (green) and nuclei counterstained with DAPI (blue) in AML12 cells treated with or without 100 μM BET for 3 h (200×; scale bar, 100 μm). The graph shows quantification of nuclear Nrf2 fluorescence intensity. Data are presented as mean ± SD and were analyzed by Student’s t-test; n = 3 independent experiments; *p < 0.05.

**Supplementary Figure 2. Betaine induces Nrf2 target genes in AML12 cells.**

(a) Immunoblot analysis of GSS, GCLM, CAT, SOD2, and HO-1 in AML12 cells treated with 0, 25, 50, or 100 μM BET for 3 h. Actin was used as a loading control. The corresponding densitometric quantification is shown. (b) Real-time qPCR analysis of *Gss*, *Gclm*, *Cat*, *Sod2*, and *Hmox1* mRNA levels in AML12 cells treated with or without 100 μM BET for 3 h. (c) Real-time qPCR analysis of *Keap1* mRNA levels in AML12 cells treated with or without 100 μM BET for 3 h. Data are presented as mean ± SD and were analyzed by Student’s t-test; n = 3 independent experiments. N.S., not significant; *p < 0.05; **p < 0.01.

**Supplementary Figure 3. Effect of chloroquine on betaine-induced Gss mRNA expression in AML12 cells.**

Real-time qPCR analysis of *Gss* mRNA levels in AML12 cells pretreated with or without chloroquine (CQ) for 24 h, followed by treatment with or without 100 μM BET for 3 h. Data are presented as mean ± SD and were analyzed by Student’s t-test; n = 3 independent experiments. N.S., not significant; *p < 0.05.

**Supplementary Figure 4. LC–MS/MS identification of betaine-associated methylation at Keap1 Cys513 and Cys518.**

Purified recombinant human Keap1 protein (5.64 μg) was incubated with 1.2 μM betaine for 1 h at room temperature in a final volume of 100 μL, followed by treatment with 60 μM DTT for 15 min. Proteolytic Keap1 peptides were then analyzed by LC–MS/MS. Representative MS/MS spectra show methylation-associated mass shifts of +14.015 Da on peptides containing Cys513 and Cys518. Fragment ions supporting the modified peptide assignments are indicated in the spectra.

**Supplementary Figure 5. Molecular dynamics simulation-based comparison of wild-type and methylated Keap1–p62 complexes.**

Annealing molecular dynamics (MD) simulations were performed to compare the conformational behavior and interaction profiles of wild-type and methylated Keap1 bound to p62. (a,c) Principal component analysis (PCA) plots showing conformational sampling of the wild-type (a) and methylated (c) Keap1–p62 complexes during the MD simulation. (b,d) Free energy landscapes projected onto PC1 and PC2 for the wild-type (b) and methylated (d) Keap1–p62 complexes. (e,f) Heatmaps showing the interaction profiles between p62 and Keap1 residues during the MD simulation for the wild-type (e) and methylated (f) complexes. (g) MM-GBSA-based binding energy calculation of the Keap1–p62 complexes, including dG_Bind, Coulomb, Solv_GB, and vdW energy components. Wild-type and methylated complexes are shown in blue and orange, respectively.

**Supplementary Figure 6. Betaine increases hepatic NADPH and GSH levels in the AAP-induced liver injury model.**

(a) Quantification of NADPH fluorescence intensity in liver sections from AAP- and AAP+BET-treated mice. (b) Measurement of hepatic GSH levels in liver tissues from AAP- and AAP+BET-treated mice. Data are presented as mean ± SD and were analyzed by Student’s t-test; n = 5 mice per group. *p < 0.05.

**Supplementary Figure 7. Serum ALT and AST levels in the AAP-induced acute liver injury model with DMG or betaine co-administration.**

(a,b) Serum ALT (a) and AST (b) levels were measured in mice assigned to CON, AAP, AAP+DMG, and AAP+BET groups. The groups are arranged in this order and shown using group-specific colors consistent with the related AAP model figures. Data are presented as mean ± SD and were analyzed by Student’s t-test for the indicated comparisons; n = 5 mice per group. N.S., not significant; *p < 0.05; **p < 0.01; ***p < 0.001; ****p < 0.0001.

**Supplementary Figure 8. Additional analyses of liver injury, inflammation, and Keap1–Nrf2 signaling in the AAP-induced liver injury model.**

Mice were orally administered BET for 7 days and challenged with acetaminophen (AAP) 2 h after the final BET dose; liver tissues were collected 12 h after AAP challenge. (a) Representative grayscale micro–CT images of mouse liver regions. Images were aligned along comparable anatomical axes, and the grayscale intensity scale indicates relative signal intensity. (b) Immunoblot analysis of IL-6 in mouse liver tissues. (c) Immunoblot analysis of CYP2E1 in mouse liver tissues. (d) Quantification of Keap1 fluorescence intensity extracted from liver immunofluorescence images. (e) Quantification of LC3B fluorescence intensity extracted from liver immunofluorescence images. (f) Co-immunoprecipitation of Keap1 from liver lysates followed by immunoblotting for p62 and Keap1; the 50 kDa band corresponds to the IgG heavy chain detected by the secondary antibody. (g) Immunoblot analysis of nuclear Nrf2 in liver nuclear fractions; GAPDH was used as a negative control for nuclear extraction. (h) Immunoblot analysis of GSS, GCLM, and GCLC in mouse liver tissues. (i) Pearson correlation analysis between Keap1 signal and MPO-positive cells in AAP-treated groups. Quantification is shown to the right where indicated. Data are presented as mean ± SD and were analyzed by Student’s t-test; n = 5 mice per group unless otherwise stated. For the correlation analysis, n = 7. N.S., not significant; *p < 0.05.

**Supplementary Figure 9. Keap1 fluorescence intensity in the MCD diet-induced MASLD model.**

Representative immunofluorescence images of Keap1 (green) and nuclei counterstained with DAPI (blue) in liver sections from CON, BET, MCD, and MCD+BET mice are shown on the left (200×; scale bar, 100 μm). Quantification of Keap1 fluorescence intensity is shown on the right. Data are presented as mean ± SD and were analyzed by Student’s t-test for the indicated comparisons; n = 5 mice per group. N.S., not significant; *p < 0.05.

**Supplementary Figure 10. Supplementary transcriptomic analysis of *KEAP1*, Kelch-domain genes, and MASLD-related pathway associations.**

(a) Co-expression pattern between *KEAP1* and *NFE2L2* in the human MASLD liver RNA-seq cohort, with dot colors representing MASLD activity score. (b) Correlation matrix showing the relationships among *NFE2L2*, *KEAP1*, MASLD severity, and Kelch-domain-containing genes associated with MASLD activity score. (c) KEGG non-alcoholic fatty liver disease pathway map highlighting genes identified by association analysis with MASLD activity score. Red stars indicate genes significantly associated with MASLD activity score.

**Supplementary Figure 11. Effect of MG132 on betaine-induced *Gss* mRNA expression in AML12 cells.**

Real-time qPCR analysis of *Gss* mRNA levels in AML12 cells treated with or without 100 μM BET in the presence or absence of MG132. Data are presented as mean ± SD and were analyzed by Student’s t-test for the indicated comparisons; n = 3 independent experiments. N.S., not significant; *p < 0.05.
